# New cyanobacterial genus *Argonema* is hiding in soil crusts around the world

**DOI:** 10.1038/s41598-022-11288-4

**Published:** 2022-05-03

**Authors:** Svatopluk Skoupý, Aleksandar Stanojković, Markéta Pavlíková, Aloisie Poulíčková, Petr Dvořák

**Affiliations:** Department of Botany, Faculty of Science, Palacký University Olomouc, Šlechtitelů 27, 783 71 Olomouc, Czech Republic

**Keywords:** Ecology, Genetics, Microbiology

## Abstract

Cyanobacteria are crucial primary producers in soil and soil crusts. However, their biodiversity in these habitats remains poorly understood, especially in the tropical and polar regions. We employed whole genome sequencing, morphology, and ecology to describe a novel cyanobacterial genus *Argonema* isolated from Antarctica. Extreme environments are renowned for their relatively high number of endemic species, but whether cyanobacteria are endemic or not is open to much current debate. To determine if a cyanobacterial lineage is endemic is a time consuming, elaborate, and expensive global sampling effort. Thus, we propose an approach that will help to overcome the limits of the sampling effort and better understand the global distribution of cyanobacterial clades. We employed a Sequencing Read Archive, which provides a rich source of data from thousands of environmental samples. We developed a framework for a characterization of the global distribution of any microbial species using Sequencing Read Archive. Using this approach, we found that *Argonema* is actually cosmopolitan in arid regions. It provides further evidence that endemic microbial taxa are likely to be much rarer than expected.

## Introduction

Biological soil crusts are pioneering communities that can form on disturbed soils, protecting them from the erosion and improving water retention^1^. They represent complex assemblages of soil particles, cyanobacteria, lichens, microfungi, bryophytes, algae, bacteria, and their exudates. Soil crust communities live on top of the soil or within the uppermost millimeters, covering the ground as a coherent layer^1^. Biological soil crusts can be found on soils all around the world, but are especially prominent in arid regions. Since arid regions make over 30% of the Earth’s surface, soil crusts have a significant impact on the global environment^1^. Well-developed soil crusts create an environment for other organisms (such as vascular plants, insects, nematodes, etc.) to flourish. They play a key role in the binding of loose soil elements and water retention, due to the production of exopolysaccharides and the formation of large colonies^2,3^. Soil crusts are dispersed globally in arid regions; in both hot and cold deserts, as well as in arid high elevation regions such as Himalaya. Soil crusts are among the most important sources of carbon and nitrogen in extreme arid habitats such as polar desserts^4^.

Soil crusts of Antarctica are dominated by the filamentous cyanobacteria (e.g., *Nostoc, Stigonema*, *Phormidium*, and *Microcoleus*)^5^. The species composition of soil crusts can differ greatly based on the local environment and geographical locality^5^. Currently, only 35 species of terrestrial cyanobacteria have been identified from Antarctica (compared to 196 species in Europe), and most of these species were isolated from soil crusts (Algaebase, as of 30th June 2021)^6^. Lambrechts et al.^7^ note that up to 85% of Antarctic soil bacterial sequences belong to uncultured genera. Antarctica, and Polar Regions in general, are especially susceptible to changes induced by global warming. The mean temperature in the Antarctic Peninsula (Western Antarctica) rose by 3 °C in the last 50 years^8^. The ice-free coastal regions of Antarctica are especially affected by environmental changes and by human activity. Currently, it remains unclear, as to how severe the effect of human activity and climate change on Antarctic microbiota will be, as the debate is still ongoing and more data are needed. The composition of cyanobacterial communities has not changed much in the last 100 years, but that might be due to the already extreme environment and the ability of Antarctic cyanobacteria to adapt to the environmental changes^9^. Nevertheless, human activity and proceeding climate change may introduce invasive species of cyanobacteria, which will be better suited for the changing climate^9^. This could have a detrimental effect on native Antarctic cyanobacterial diversity. Thus, it is important to investigate the diversity of biological soil crusts in the rapidly changing Antarctic environment while we still have a chance.

The diversity of cyanobacteria has been most thoroughly studied in temperate regions, especially in Europe and Northern America^10–12^. However, there are regions where the diversity of soil cyanobacteria remains largely unexplored, especially tropical^11^ and polar regions^13^. This difference is likely due to the under-sampling, as a majority of cyanobacteriological studies focus on temperate regions and plankton^11^. Moreover, only ~ 20% of species in the GenBank are correctly identified and at least half of cyanobacterial species have never been successfully cultured^14^. Therefore, the diversity of cyanobacteria seems to be largely unexplored. Many taxa have been described as endemic (e.g. *Kastovskya adunca*^15^), but, in other cases, a more thorough exploration might uncover wider distribution^16^.

Historically, cyanobacterial diversity was studied using morphological and ecological data. However, morphological features may be plastic and dependent on conditions of cultivation, such as light, temperature, and composition of the medium. Perhaps, the biggest issue of morphological determination is a high level of cryptic diversity in cyanobacteria^17^. Distant clades of cyanobacteria can have very similar or even indistinguishable phenotypes, leading to polyphyly and species miss-identification. Cryptic diversity can occur between closely related species but also between distantly related genera. For instance, the simple coccoid genus *Synechococcus* contains at least 12 polyphyletic lineages that cannot be distinguished based on the morphology. Cryptic diversity in cyanobacteria is caused mainly by serial convergent evolutionary events, which is projected to taxonomy by the observation that the majority of described cyanobacterial genera are actually polyphyletic^17,18^. Thus, a combination of all available evidence composed of molecular, morphological, and ecological data is used for the taxa delimitation; often referred to as the polyphasic approach^19^.

The majority of newly proposed species and genera of cyanobacteria are described based on 16S rRNA phylogeny. However, the use of the 16S rRNA alone is often insufficient to distinguish taxa at or below the species level^20^. Some diversity can only be understood by using the whole genome^19^. In recent years, the decreasing price of whole genome sequencing (WGS) has made it more accessible and allowed for its use in phylogeny^21^. WGS can resolve much finer patterns of diversity at and below the species level For example, WGS has been employed to describe the tropical cyanobacteria *Ellainela*^22^ and *Moorena*^23,24^.

The relatively low cost of sequencing has also allowed for expanding employment of metagenomics to aid in the study of microbial diversity and distribution. For example, metagenomic data has been obtained from sampling studies from soils around the world^25^, providing an abundant source of information on cyanobacterial diversity and distribution that remains largely untapped. Taxonomic studies do not often focus on the distribution because strain isolation is laborious and time-consuming. Most of the studies that focused on patterns of distribution use available taxonomic databases, where new taxa are added with a relatively long delay and they contain many erroneous identifications^14^. Moreover, it is way too expensive to make a sampling expedition for every new taxon. Unfortunately, the importance of taxonomy has been diminished and funding is scarce, although it is fundamental to all branches of biology^26^. Here, we provide a framework for mining of the distributional data from existing metagenomic databases. We were able to connect the genome sequence of the type material with a wealth of sequencing data available. Thus, we propose a mechanism for the integration of metagenomic and whole genomic approaches to erect new taxa and uncover patterns of biogeographic distribution.

The family Oscillatoriaceae (sensu Komárek et al.)^18^ includes some of the most well-known genera (e.g., *Oscillatoria*) and it is one of the largest families with 861 species (Algaebase as of 30th June 2021) of freshwater, marine, and terrestrial taxa. However, this family was historically used as a tentative group, holding virtually any cyanobacteria with simple filamentous morphology. With the advent of modern molecular methods, many of the genera were identified as polyphyletic, including some of the most common, species-rich genera (e.g. *Phormidium*^27^ and *Lyngbya*^28^). This group is thus in dire need of taxonomic revisions. The epitype of *Oscillatoria* has been established recently by Mühlsteinová et al.^29^ (CBFS A-89-1, *O. princeps* CCALA 1115), but many other genera in this family are yet to be discovered.

The goal of this study is to advance the understanding of the biodiversity of terrestrial cyanobacteria. We studied 12 cyanobacterial strains from Antarctica and used a combination of morphological, phylogenetic, and genomic data to describe a new genus and two new species of filamentous terrestrial cyanobacteria. Furthermore, we aimed to develop a method to search the Short Read Archive and assess the distribution of this new genus.

## Results

*Argonema* gen. nov. Skoupý et Dvořák.

Type species: *Argonema galeatum.*

Morphology: Filamentous cyanobacterium, colonies macroscopic, growing in round bulbs and tufts. The filaments are dark green to blue-green, grey-green or brown-green in color. Cells are wider than they are long. Filaments sheathed, sheaths are colorless to light brown, distinct, and variable in length. The filament can protrude from the sheath or the sheath can exceed filament. Trichomes are cylindrical, not attenuated to slightly attenuated towards the end, slightly or not constricted at cell walls. The apical cell can be concave, dark brown, purple-brown to almost black. Cell content often granulated. Necridic cells present, reproduction by hormogonia. The morphological description was based on both culture and fresh material.

Etymology: The genus epithet (*Argonema*) is derived from greek *Argo* – slow, latent (αργός) and *nema* – thread (νήμα).

*A. galeatum* sp. nov. Skoupý et Dvořák.

**Figures 1-8:**
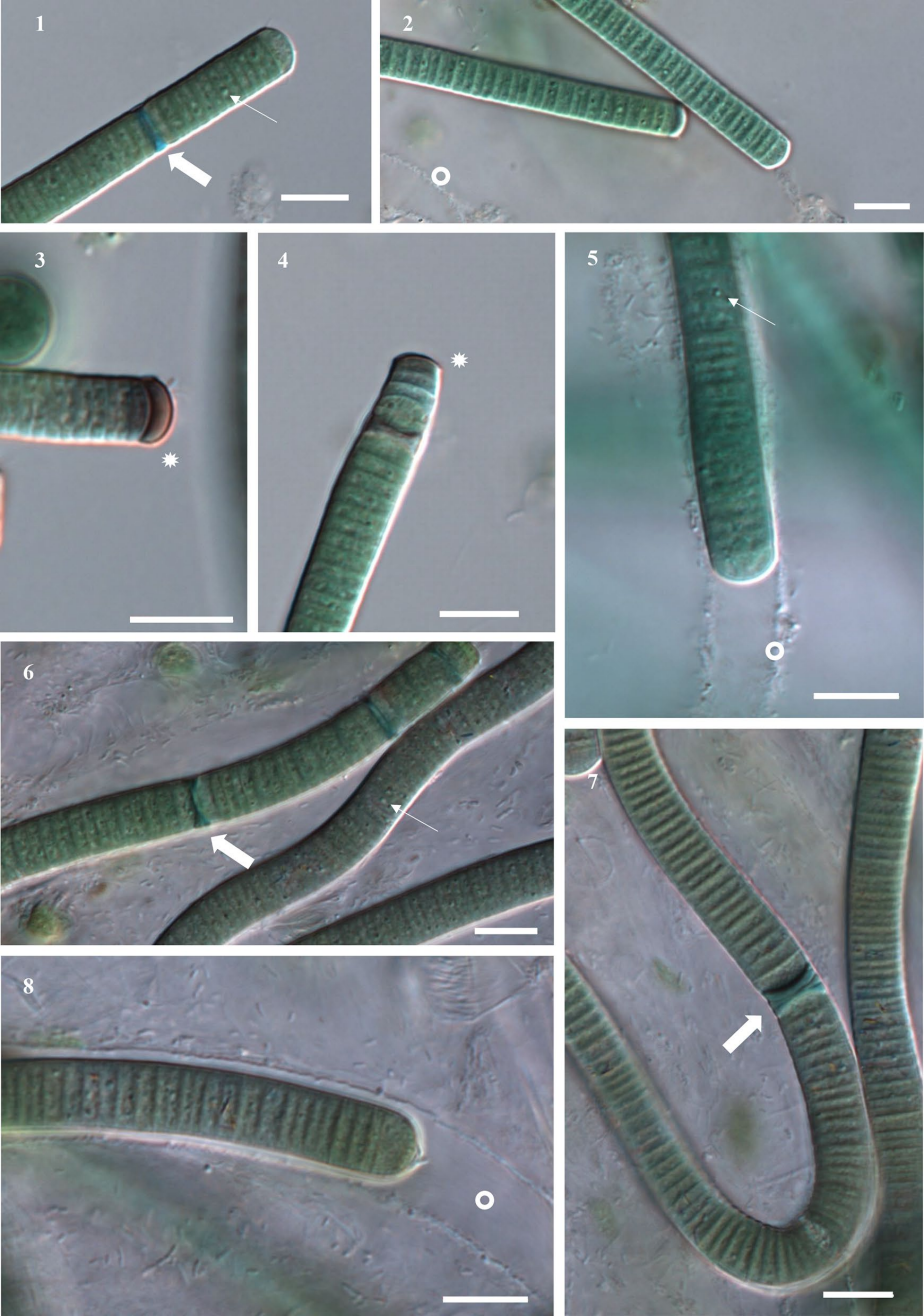
Microphotographs of *Argonema galeatum* (Figs 1–5) and *Argonema antarcticum* (Figs. 6–8) Trichomes of *A. galeatum* appear more straight (Fig 2), while trichomes of *A. antarcticum* form waves (Fig 6) and loops (Fig 7). Scale = 10 µm, wide arrow = necridic cells, arrowhead = granules, asterisk = colored apical cell, circle = empty sheath.

Morphology: The cells of *A. galeatum* are 6.5–9.1 µm (mean 7.81 µm) wide and 1.1–2.5 µm (mean 1.83 µm) long (Figs. 1–5). Filaments are straight, blue-green to gray-green in color. The sheaths are colorless to light brown, distinct, and variable in length. The filament can protrude from the sheath or the sheath can exceed filament. No true branching was observed. Trichomes are cylindrical, not attenuated or slightly attenuated towards the end, slightly or not constricted at cell walls. Some filaments have a concave apical cell that is dark brown, purple-brown to almost black (Fig. 11b). Cell content often granulated. Reproduction by necridic cells and subsequent breaking of the filaments into hormogonia (Fig. 11a,c). The morphological description was based on both culture and fresh material.

**Figures 9 and 10:**
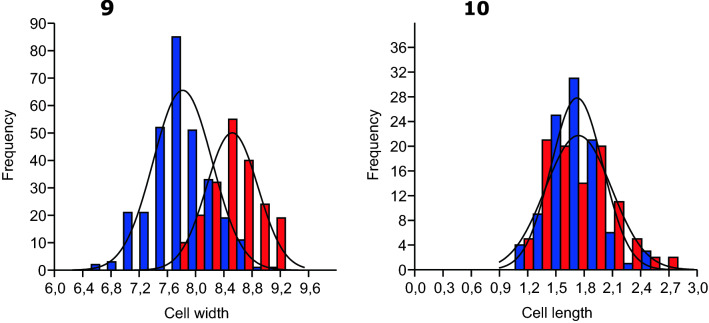
Histograms of cell dimensions constructed using PAST software. Fig. 9 – Histogram of cell width frequencies in *A. galeatum* (blue) and *A. antarcticum* (red). Fig. 10 – Histogram of cell length frequencies in *A. galeatum* (blue) and *A. antarcticum* (red).

Holotype: 38,057, Herbarium of the Department of Botany (OL), Palacký University Olomouc, Czech Republic.

Reference strain: *Argonema galeatum* A003/A1.

Type locality: James Ross Island, Western Antarctica, 63.80589S, 57.92147 W.

Habitat: Well-developed soil crust.

Etymology: Species epithet *A. galeatum* was derived from latin *galea* – helmet.

*A. antarcticum* sp. nov. Skoupý et Dvořák.

**Figure 11:**
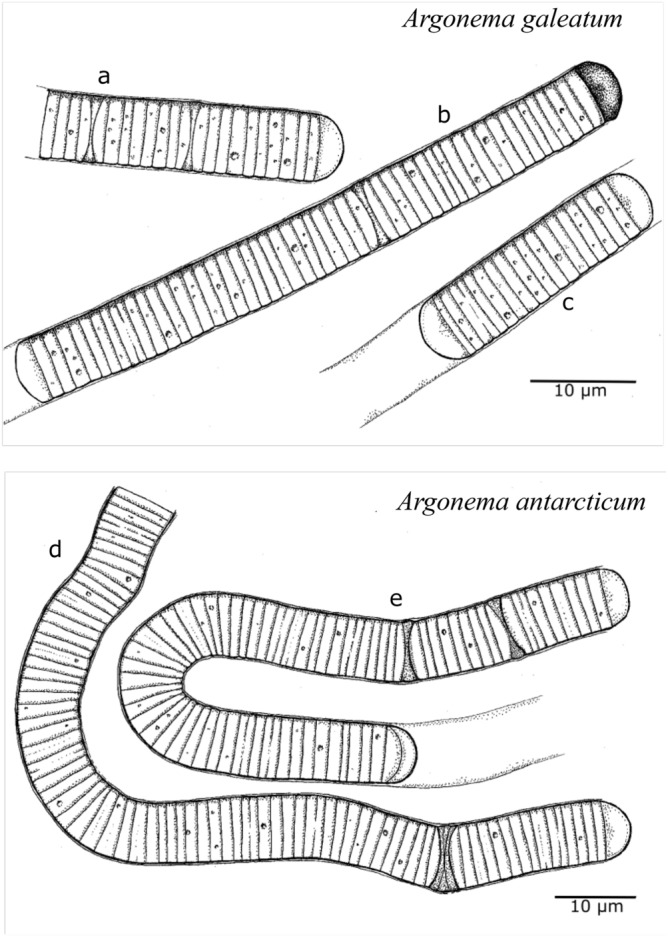
Lineworks of *Argonema galeatum* (**a**–**c**) and *Argonema antarcticum* (**d**, **e**). (**b**) – filament of *A. galeatum* with typical dark apical cell. (**c** – *A. galeatum* hormogonium. (**e**) – filament of *A. antarcticum* with necridic cells and protruding sheath.

Morphology: The cells are 7.6–9.2 µm (mean 8.52 µm) wide and 1.2–2.8 µm (mean 1.72 µm) long (Figs. 5–8). Filaments are wavy, gray-green to brown-green in color. The sheaths are colorless to light brown, distinct, and variable in length. The filament can protrude from the sheath or the sheath can exceed filament. No true branching was observed. Trichomes are cylindrical, not attenuated or slightly attenuated towards the end with a concave apical cell, slightly or not constricted at cell walls (Fig. 11d). Necridic cells present (Fig. 11e), reproduction by hormogonia. The morphological description was based on both culture and fresh material.

Holotype: 38,058, Herbarium of the Department of Botany (OL), Palacký University, Olomouc, Czech Republic.

Reference strain: *Argonema antarcticum* A004/B2.

Type locality: James Ross Island, Western Antarctica, 63.89762S, 57.79743 W.

Habitat: Well-developed soil crust.

Etymology: Species epithet *A. antarcticum* was derived from the original sampling site.

### Morphological variability

We used light microscopy to assess the morphology of *Argonema* from soil crust samples and cultured strains. *Argonema* is morphologically similar to other Oscillatoriales, such as *Lyngbya, Phormidium*, and *Oscillatoria*. In culture, the morphology of *A. galeatum* and *A. antarcticum* differed slightly. Filaments of *A. antarcticum* are wider than cells of *A. galeatum*, averaging at 8.52 µm (*A. galeatum* – 7.81 µm). The average cell width/length ratio is 4.54 for *A.galeatum* and 4.89 for *A. antarcticum*. The cell width was significantly different between the two species (Nested ANOVA, *p* < 0.0001; Fig. 9). Cell length is less variable between the two species, with both averaging at 1.7 µm (Fig. 10). The difference in cell length was not statistically significant (Nested ANOVA, *p* = 0.7261). The distinctly colored concave apical cell was observed only in *A. galeatum* strains (Fig. 11). No true branching and no aerotopes were observed in either species. We also observed the morphology of uncultured filaments in the native sample. These filaments matched the morphological characteristics of cultured *Argonema* strains (Supplements, Fig S1).

### Phylogeny

**Figure 12:**
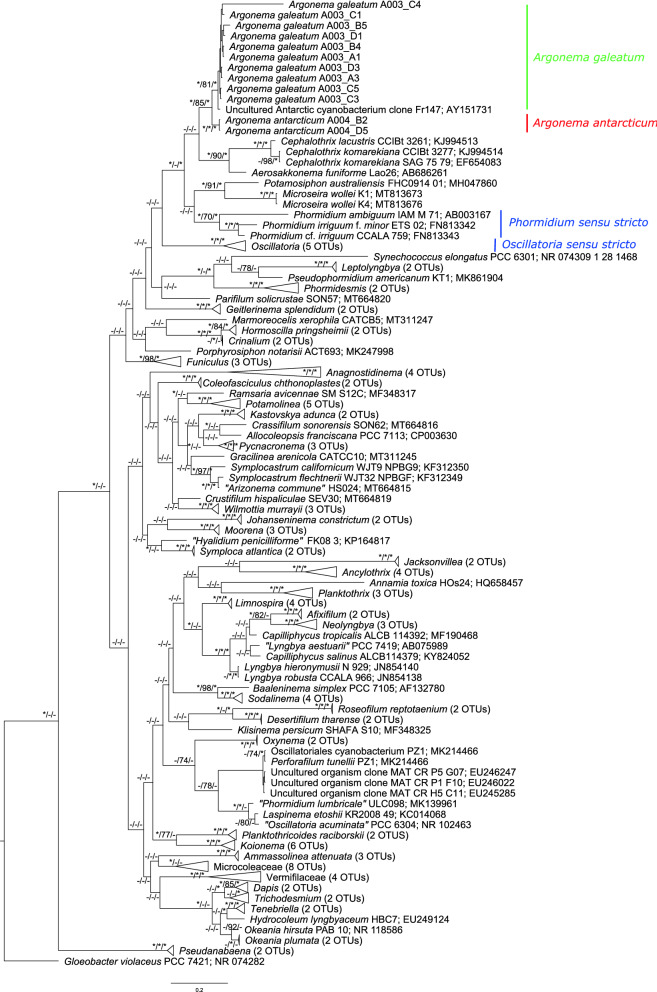
Phylogenetic reconstruction of *Argonema* based on 16S rRNA. The phylogenetic tree was constructed using Bayesian inference. Node labels represent node supports; Bayesian inference/maximum parsimony/maximum likelihood. Nodes with a support of 99 or 100 are marked with a star and supports below statistical significance are marked with a dash.

Phylogeny based on the 16S rRNA gene using Bayesian inference, maximum likelihood, and maximum parsimony revealed that strains of *Argonema* form a distinct monophyletic clade among other Oscillatoriales (Fig. 12). The A003 strains formed one clade, while the two A004 strains formed a highly supported sister clade, suggesting that they may be different species. The A003 clade also contains an uncultured Antarctic cyanobacterium clone AY151731^30^. The closest clade to that formed by *Argonema* lineages was a clade containing *Cephalothrix komarekiana* and *Aerosakkonema funiforme* (Fig. 12). The second closest clade contained *Potamosiphon australiensis*, *Microseira wollei*, *Phormidium irriguum*, and *Phormidium ambiguum*. *Argonema* formed a distinct clade different from *Oscillatoria sensu stricto* and *Phormidium sensu stricto*^31^.

There are two significant insertions in the 16S-23S ITS present in *A. antarcticum* strains that are 9 bp and 6 bp long (Supplements, Fig. S2). We also estimated the secondary structure of D1-D1-’ and Box B helices in 16S-23S ITS. The secondary structures of *A. galeatum* and *A. antarcticum* were identical (Supplements, Fig. S3).

**Figure 13:**
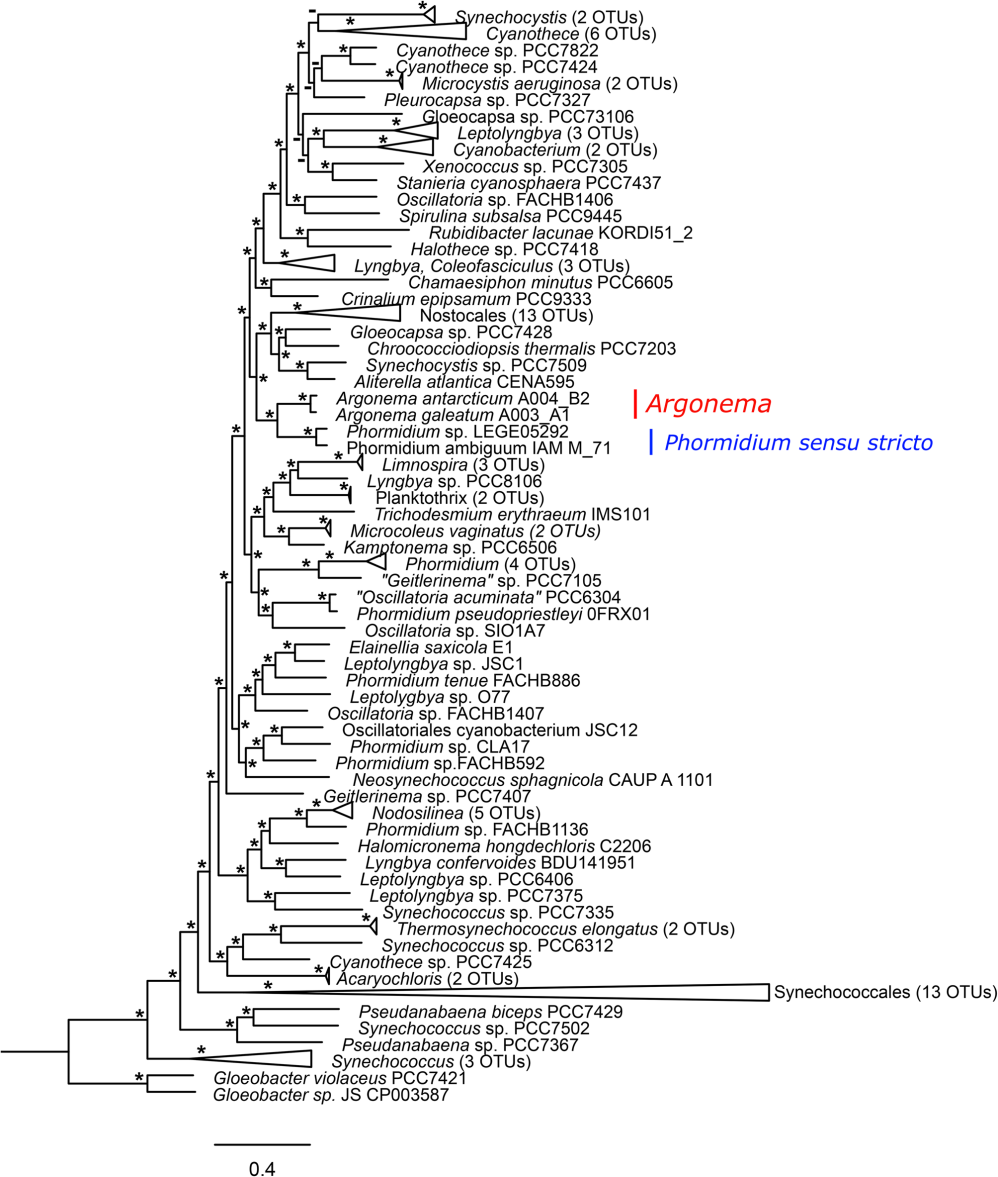
Maximum likelihood phylogenomic reconstruction of cyanobacteria based on 153 585 amino acid sites. Nodes with bootstrap support of 99 or 100 are marked with an asterisk.

16S rRNA sequence may not often be sensitive enough for a species delimitation in cyanobacteria^20^, so we investigated the whole-genome sequence for each putative species. Two strains were selected for whole genome sequencing, one *A. galeatum* strain (A003/A1) and one *A. antarcticum* strain (A004/B2). Both strains have average genome sizes similar to other filamentous cyanobacteria (6–8 Mb)^32,33^. Both genomes have very similar and average GC content, but differ slightly in the number of coding sequences and number of RNAs (Table 1). 117 annotated cyanobacterial genomes were obtained from the NCBI database for phylogenomic reconstruction (Fig. 13). Both strains of *Argonema* clustered with genomes of *Phormidium* sp. LEGE05292 and *Phormidium ambiguum* (strain IAM M-71) as in 16S rRNA phylogeny. *Argonema* thus belongs to the Oscillatoriales order, to the family Oscillatoriaceae *sensu* Komárek et al.^18^.

**Table 1:** Basic genome properties of *Argonema galeatum* strain A003/A1 and *Argonema antarcticum* strain A004/B2.

Genome properties	*A. galeatum* A003/A1	*A. antarcticum* A004/B2
Size	6 929 784 bp	7 745 032 bp
GC Content	44.9%	44.5%
N50*	112,421 bp	52,657 bp
Number of coding sequences	7369	8215
Number of RNAs	69	75

*Sequence length of the shortest contig at 50% of the total genome length.

We used whole genome average nucleotide identity as additional evidence for erecting a new species. The ANIb score of A003/A1 *A. galeatum* and A004/B2 *A. antarcticum* was 92.16–92.55, the ANIm score was 93.73–93.74. Both of these scores are lower than the 95–96% threshold for species delimitation^34^. The scores between A003/A1 *A. galeatum* and *Phormidium ambiguum* IAM M-71 were 71.03–71.23 (ANIb) and 83.67–83.63 (ANIm) and the scores between A003/A1 *A.galeatum* and *Phormidium* LEGE05292 were 71.4–71.62 (ANIb) and 83.9–83.98 (ANIm). The scores between A004/B2 *A. antarcticum* and *Phormidium ambiguum* IAM M-71 were 71.73–71.86 (ANIb) and 85.68–85.73 (ANIm), the scores between A004/B2 *A. antarcticum* and *Phormidium* LEGE05292 were 71.85–71.72 (ANIb) and 85.29–85.42 (ANIm). (Supplements, Table S1).

Taken together with a morphological difference, this evidences that we recognize two species within *Argonema* – *A. galeatum* and *A. antarcticum.*

We also calculated average percent similarity between *Argonema galeatum* and *Argonema antarcticum* strains and closely related strains, based on 16S rRNA phylogeny (Supplements, Table S2). The average percent similarity between *A. galeatum* and *A. antarcticum* strains was 98.7%, while the similarity within the two *Argonema* clades was 99% and higher. One exception was an *Argonema galeatum* strain A003/C4, which had only approximately 94.8 average percent similarity to other *Argonema* strains. This was likely due to the lower quality of this 16S rRNA sequence.

### Distribution

**Figure 14:**
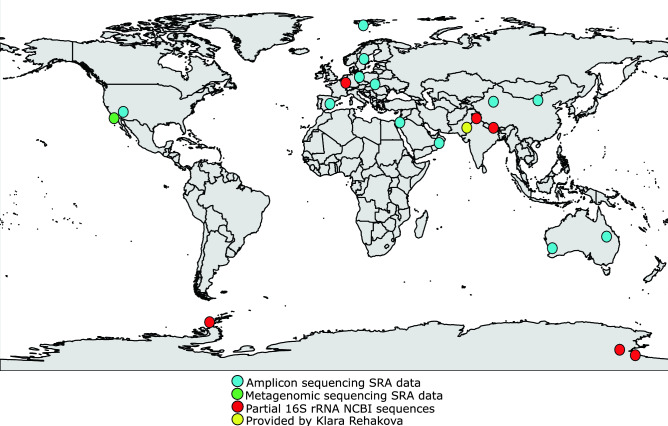
Geographical distribution of *Argonema* genus based on metagenomic data. The map was constructed using R software (www.R-project.org), with packages rnaturalearth v0.1.0 (http://github.com/ropenscilabs/rnaturalearth) and ggplot2 v.3.3.5 (https://ggplot2.tidyverse.org), and modified in Inkscape (http://www.inkscape.org).

To investigate the distribution of *Argonema*, we searched the Sequence Read Archive (SRA, https://www.ncbi.nlm.nih.gov/sra) using 1937 amplicon, 223 RNA, and 191 whole metagenome datasets (1.7 TB of data). We found matches to *Argonema* in 37 amplicon, 4 whole metagenome datasets, and no match in RNA datasets (Supplements, Table S3). Matches with amplicon sequencing were considered positive if at least one 16S rRNA sequence with 97% or higher identity and read length over 100 bp was recovered. Positive matches from metagenomics sequencing were considered if the genome coverage was covered by reads at least 10x. The datasets belonged to uncultured cyanobacteria from soil samples or soil crusts from diverse geographic locations, most notably USA (California, Utah, New Mexico, and the Mojave Desert), China (Tengger Desert, Tibetan Plateau, Gurbantunggut Desert), and Israel, but also Svalbard, Antarctica, Spain, Germany, Austria, Australia, India, and Oman (Fig. 14).

We also searched the NCBI nucleotide database for partial sequences of 16S rRNA of uncultured cyanobacteria similar to *Argonema*, and constructed a maximum likelihood tree with sequences with 97% or more similarity to *Argonema* (Supplements, Fig. S4). We found 15 short partial 16S rRNA sequences in the NCBI database (< 413 bp). These sequences also came from diverse geographical locations (Supplements, Table S3), mostly from Antarctica, notably from McMurdo Dry Valleys, but also from Nepal, Pakistan, and even Luxembourg (Fig. 14).

In total, we discovered 57 possible *Argonema* matches at 57 geographical localities. Samples were generally collected from soils or soil crusts, but there were also samples collected from melt-water lakes in Antarctica and also one from a freshwater lake (Luxembourg). Samples came from geographical areas with annual precipitation lower than the global average (950 mm), with most samples from areas with annual precipitation lower than 500 mm (Supplements, Table S3).

### Discussion

The diversity of soil cyanobacteria remains largely unexplored. Many taxa have been erected in the last decade, but their geographical distribution is still largely unknown outside of type locality. In this study, we describe a new genus of cyanobacteria with two species. This genus was firstly found on James Ross Island, Western Antarctica, but we show that it has a cosmopolitan distribution in soils and soil crusts. Our findings stress that our knowledge of the diversity of microbes is still very limited. We provide evidence that the concept of endemism in microbes is heavily dependent on a sampling effort, even in extreme environments such as Antarctica^35^. Moreover, sequence data archives represent a wealthy source of data available to study the distribution of newly discovered microbes.

*Argonema* forms a distinct and highly supported clade among other Oscillatoriales, based on both phylogenetic (16S rRNA) and phylogenomic data. Phylogeny based on whole genomes clustered *Argonema* strains as a sister clade to a clade consisting of *Phormidium ambiguum* and *Phormidium irriguum*, which belong to the genus *Phormidium *sensu stricto according to Komárek^31^. Currently, no whole-genome sequence is available for either *Cephalothrix*, *Aerosakkonema*, *Potamosiphon* or *Microseira*, thus these genera could not be included in the phylogenomic analysis. Based on the phylogenomic and phylogenetic analysis, *Argonema* genus belongs to the order Oscillatoriales, to the family Oscillatoriaceae, sensu Komárek et al.^18^.

Based on 16S rRNA phylogeny, we recognize two separate clades in *Argonema* representing two species: *A. galeatum* and *A. antarcticum*. Additional evidence supporting a division of *Argonema* into two species was provided by ANI values estimation^36^. Both the ANIb and ANIm scores between *A. galeatum* and *A. Antarcticum* were lower than the 95–96% threshold for species delimitation. Interestingly, the genomes of *A. galeatum* and *A. antarcticum* were very similar in GC content, the number of genes, and overall size. In cyanobacteria, genome size and GC content are often connected with adaptations to a new habitat or environment^33,37^, so the similar GC content and genome size in *Argonema* species are likely a result of adaptation to the same environment. Further evidence supporting the division of *Argonema* strains into two species is the presence of 2 significant insertions/deletions in the 16S-23S ITS gene (Supplements, Fig. S2). We also calculated the average percent similarity between *Argonema galeatum* and *Argonema antarcticum* strains and closely related strains, based on 16S rRNA phylogeny (Supplements, Table S2). Generally, the average percent similarity between *A. galeatum* and *A. antarcticum* was quite high, at 98.7%. We believe, however, that the division of *Argonema* strains into two species is sufficiently supported by other evidence.

The two proposed species *A. galeatum* and *A. antarcticum* can be differentiated based on their 16S rRNA phylogeny, whole genome phylogeny, and morphological features. Compared to other prokaryotes, cyanobacteria could possess sufficient variability of morphological traits which can be used for identification. Thus, we performed a detailed analysis of the *Argonema* strains morphology. Some filaments of *A. galeatum* possess distinct colored apical cells, which were not observed in *A. antarcticum*. The distinct apical cell may play a role in burrowing of the trichomes into the substrate as in other soil filamentous cyanobacteria such as *Microcoleus vaginatus*^38^. It is currently unclear, whether the apical cell is dark colored itself, or if it is covered in dark structure, e.g. apex of a sheath. Filaments of *A. galeatum* were generally straight, whereas filaments of *A. antarcticum* formed waves and loops. Trichomes of *A. antarcticum* are significantly wider than those of *A. galeatum*. *A. antarcticum* also differed in color from *A. galeatum*, with trichomes being more brown-green to gray-green, rather than blue-green.

*Argonema* is morphologically similar to other Oscillatoriales, but it can be differentiated from its closely related genera based on morphology. *Argonema* can be distinguished from *Ceplhalothrix* based on the absence of aerotopes in cells. Trichomes of *Cephalothrix komarekiana* are also narrower, at 4.8–7.3 µm wide. Apical cells of *C. komarekiana* trichomes can be capitate and calyptras can be present. The apical cells of *Argonema galeatum* can be distinctly colored, but no calyptras were observed in either *A. galeatum* or *A. antarcticum*. Also, while *Argonema* is a predominantly soil cyanobacterium from areas with generally low precipitation, *Cephalothrix* genus is found in a tropical or sub-tropical freshwater environment. The type species, *Cephalothrix komarekiana* is a tropical freshwater species isolated from Brazilian Pantanal Wetlands^39^. *Aerosakkonema funiforme* is also a freshwater cyanobacterium isolated from the mesotrophic reservoir in the Lao People's Democratic Republic^40^. *Aerosakkonema* can also be differentiated from *Argonema* by the presence of small aerotopes in cells. Trichomes of *Aerosakkonema funiforme* are wider, at 11.7–16.6 µm wide (compared to avg 6.5–9.2 µm of *Argonema*), and do not have sheaths. *Argonema* can also be morphologically and ecologically differentiated from *Potamosiphon australiensis* and *Microseira wollei*. *Potamosiphon australiensis* was originally isolated from benthos of the freshwater stream in Australia^41^. Filaments of *P. australiensis* are cylindrical, approximately 20–22 µm wide, not or slightly constricted at cell walls, and encased in distinct and sometimes lamellated sheaths. Two or more filaments often share one sheath following diagonal division. *P. australiensis* reproduces via motile hormogonia, which often form in series. *Microseira wollei* is a mat-forming cyanobacterium described from freshwater environments in Australia^42^. Filaments of *M. wollei* are significantly wider than filaments of *Argonema*, 30–65 µm wide, and encased in a distinct lamellated sheath. False branching was also observed in *Microseira*, which was not observed in *Argonema*. Distinctly colored apical cells were not observed in either *Potamosiphon* or *Microseira*.

*Argonema* can also be morphologically differentiated from *Phormidium ambiguum* and *Phormidium irriguum*, which belong to the *Phormidium *sensu stricto^31^. *P. ambiguum* was described in 1892^43^ from northern Germany as a freshwater/marine species, bright blue-green to yellow-green in color, with trichomes 4.5–7.5 µm wide, not attenuated, not capitate, with thin distinct sheaths. However, Compére^44^ described a variant *P. ambiguum* var. *major* that has wider trichomes at 9.5 µm wide, which are shortly attenuated towards the end and have a calyptra. Currently, there is a partial 16S sequence (AB003167)^45^ and a whole genome sequence of *P. ambiguum* available in the NCBI database (strain IAM M-71)^46^, but there are no morphological data available to assess which morphological variant it is. *Phormidium irriguum* is a similar case as several morphotypes differ significantly. Anagnostidis & Komárek^47^ described *P. irriguum* as blue-green or grayish in color, with cells 6–11.2 µm wide and 4–11 µm long. Apical cells are convex, slightly capitate, and with thickened cell walls. Sciuto et al.^48^ described two variants of *P. irriguum*. One CCALA 759, which has cells that are 9–12 µm wide and 2–3 µm long, and second strain ETS-02 with 3–5 µm wide and 1–2 µm long cells, trichomes with rounded apical cells, and colorless sheaths. Neither of these match in description *P. irriguum* as described by Anagnostidis & Komárek^47^. Furthermore, the partial sequence of 16S rRNA gene for *P. irriguum* CCALA 815, annotated by Strunecký et al.^49^, bears a high identity (99.24%) with a sequence of 16S rRNA of *Tychonema bourrellyi* LT546478, annotated by Salmasso et al.^50^, which is described as having trichomes 4–6 µm wide, purple or red-brown in color^47^. In this case, it might be a result of misidentification of *P. irriguum* as *T. bourrellyi* by Salmaso et al.^50^. Both *P. ambiguum* and *P. irriguum* also differ from *Argonema* in ecological requirements, as *P. irriguum* was first isolated from mossy rock surface in Switzerland^47^ and *P. ambiguum* is marine/freshwater.

No previously described species morphologically or ecologically match *Argonema*. The most similar morpho-species is *Oscillatoria subproboscidea*^51^, which was described from Antarctic coast lakes. *O*. *subproboscidea* is quite similar to *Argonema* strains in cell dimensions (cells 8.2–9 µm wide and 3–4 µm long). It was described as having suddenly attenuated and frequently uncinated, never capitate filaments. We observed some filaments that were suddenly attenuated in *Argonema* strains, especially in native samples, but only rarely. McKnight et al. ^52^ also described two morphotypes of *O*. *subproboscidea*, one that had filaments 7.5–10 µm wide with calyptrate apical cells and sheaths, and second morphotype that was 9–12 µm wide with numerate granules and possible aerotopes. While the first morphotype described by McKnight et al. could be morphologically similar to *Argonema*, the second is likely a completely different filamentous cyanobacterium^52^. In Nadeau et al.^53^, *O*. *subproboscidea* is mentioned as *Phormidium subproboscideum* and there is even a partial 16S rRNA sequence available in the database (*Phormidium* sp. Ant-brack-3, AF263332). However, the p-distance value between *Argonema galeatum* strain A003/A1 and *Phormidium* sp. Ant-brack-3, as computed by MEGA X software was 0.08 (92% similarity), which further supports the hypothesis that it belongs to a different cyanobacterial clade. Broady & Kibblewhite^54^ described several morphotypes of Antarctic *Phormidium* strains, one of which is morphologically very similar to *Argonema galeatum*, as it is described as having cells 8.2–10.9 µm wide and 2–6 µm long with sheaths and distinct apical cells. This strain was also described as *Phormidium subproboscideum* by Broady & Kibblewhite^54^, but there are no molecular data available on this strain. It is possible that *Oscillatoria subproboscidea* (or its alternative *Phormidium subproboscideum*) is a species belonging to *Argonema* genus. *Oscillatoria subproboscidea* was described more than 100 years ago and there are no genetic data available to assess its relatedness to our strains based on molecular phylogeny.

Other notable morphologically similar species is for example *Oscillatoria annae*^55^ that is described as having dull green trichomes, cells isodiametric, 7.5–8 µm wide and 1.5–3 µm long. Trichomes straight at the end, apical cells conically narrowed, rounded, and without calyptras. No colored apical cells, distinct necridic cells or distinct sheaths were observed in *O. annae* and unlike *Argonema*, which is terrestrial, *O. annae* was described as freshwater benthic cyanobacterium from temperate and tropical regions. Another morphologically similar species is *Oscillatoria tenuis* var. *levis*^56^ which is described as having straight trichomes blue-green to purple gray in color, irregularly granulated, not or slightly constricted at crosswalls, with cells 6–11 µm long and 2–3.5 µm wide. *O. tenuis* can have apical cells with a thickened outer cell wall, but no sheaths or distinct necridic cells were observed. *O. tenuis* was also described as predominantly benthic freshwater cyanobacterium from tropical regions, although possibly cosmopolitan. Another possibly similar cyanobacterium is *Lyngbya antarctica* which was described by Gain^57^ as having pale brownish to blue-green filaments with distinct sheaths and cells 7.5–9 µm wide and 1–1.5 µm long, with calyptrate apical cells. This taxon is, however, problematic. It was discovered from Antarctica, but it was only described once by Gain and there are no further records or data available of this species so its taxonomic status is uncertain.

We also analyzed the secondary structures of 16S-ITS of *A. galeatum* and *A. antarcticum*, to further support the hypothesis that they belong to two distinct species. Although the secondary structures turned out identical, we have extensive evidence based on morphological, phylogenetic, and phylogenomic reconstructions to differentiate *A. galeatum* and *A. antarcticum*.

We searched the NCBI SRA and nucleotide databases for partial sequences similar to our *Argonema* sequences to assess a possible distribution of *Argonema* genus. Our approach allows sequence database mining without previous knowledge of the taxonomic diversity within metagenomic samples. No taxonomic scheme (SILVA, https://www.arb-silva.de/; NCBI, https://www.ncbi.nlm.nih.gov/; RDP, https://rdp.cme.msu.edu/) needs to be applied. We have only limited knowledge about the distribution of most of the cyanobacterial taxa erected based on molecular data within the last two decades. Using this method, the distribution of new taxa can be evaluated upon the description, which provides a rich source of information about the importance of a particular organism. The only disadvantage is that the raw reads in the NCBI SRA cannot be efficiently searched via online form. The data must be downloaded locally. The search also produces large files. Although the files can be compressed, they still occupy terabytes of memory.

We discovered multiple sequences of *Argonema* from several soil or soil crust metagenomic samples from various geographical locations (e.g., Antarctica, China, USA, Israel, Svalbard, etc.). This indicates that *Argonema* is not endemic to Antarctica, but might be in fact cosmopolitan, only it was not properly described before. The annual precipitation in the sampling locations where the sequences of potential *Argonema* strains were obtained was generally lower than the 990 mm global average^58^, with McMurdo Dry Valleys only receiving less than 10–50 mm of precipitation annually. However, there were some exceptions with samples from Germany, Austria or Luxembourg, which have precipitation levels close to the global average. This indicates that *Argonema* might be generally associated with dry to very dry habitats and it is well adapted to low levels of precipitation or that there is an intraspecies variability, which cannot be uncovered using this approach. In environmental samples of soil crusts, *Argonema* filaments might have been overgrown by faster growing Oscillatoriales cyanobacteria, which might be one of the reasons why it avoided detection. This would be in line with the well-known Baas Becking hypothesis that “everything is everywhere but the environment selects”^59^. The obtained sequences from metagenomic studies were only partial, so it is not possible to deduce whether they belong to one of the two species described here, or if they belong to another new species in the novel *Argonema* genus.

In conclusion, we used a complex approach based on molecular, morphological, and metagenomic data, to describe a novel genus of crust-forming filamentous cyanobacteria with a potentially cosmopolitan distribution. Moreover, our findings provide evidence that the concept of endemic taxa in microbes can be much rarer than previously expected. With enough effort, the species can be found somewhere in the sea of the sequencing data.

## Materials and methods

### Strain isolation

Samples were collected in Bohemian stream valley and Solorina Valley on James Ross Island, Western Antarctica (Table 2) from well-developed soil crusts. The original sample was obtained by Michal Zeman (Masaryk University Brno, Czech Republic) on March 6 and 9 2020. We isolated 12 strains from the fresh samples using standard isolation techniques^60^. The isolated strains were grown on Zehnder medium^61^ under the following conditions: temperature 22 ± 1 °C day temperature, 18 ± 1 °C night temperature, illumination 20 µmol photons m^−2^.s^−1^, light regime 16 h light/8 h dark.

**Table 2:** Sampling localities.

	A003	A004
State/province	Western Antarctica, James Ross Island	Western Antarctica, James Ross Island
Locality	Bohemian stream valley,	Solorina Valley
Habitat	Soil crust	Soil crust
Elevation	34 m.a.s.l	154 m.a.s.l
GPS position S	63.80589	63.89762
GPS position W	57.92147	57.79743
Date of sampling	6.3.2020	9.3.2020

### Morphology assessment

We observed the morphology of all 12 strains and selected 5 strains for the morphology assessment (three A003 strains and two A004 strains) using light microscopy. Zeiss AxioImager microscope with high resolution camera AxioCam HRc 13MPx was used for morphology assessment. The following features of cultured strains were assessed: cell shape, cell dimensions, terminal cells, reproduction, sheaths, branching, and granulation. To assess the cell dimensions, the width and length of 100 cells from each observed sample were measured. Morphological data were analyzed using PAST software^62^. A nested ANOVA test was used to identify whether the morphological difference between the strains was statistically significant. We also observed the native sample and studied the morphology of uncultured filaments using the same method as with the cultured strains.

### PCR amplification and sequencing

Genomic DNA was extracted from fresh biomass (approximately 50 mg) using DNeasy UltraClean Microbial Kit (QiaGEN, Hilden, Germany), following the manufacturer´s manual. The quality of extracted DNA was checked by agarose gel electrophoresis (1.5% agarose gel, GelRed – Biotium, California, USA). Extracted DNA was quantified using NanoDrop 1000 (Thermo Fisher Scientific, Wilmington, Delaware, USA). A partial 16S rRNA sequence and whole 16S-23S ITS sequence were obtained using PCR amplification. Primers forward P2 (5´-GGGGAATTTCCGCAATGGG-3´) and reverse P1 (5´CTCTGTGTGCCAGGTATCC-3´) were used for PCR amplification. The PCR reaction was performed in a total volume of 40 µl (17 µl sterile RNAse free water, 20 µl EmeraldAMP (Takara Bio Europe SAS, Saint Germain en Laye, France) 1 µl P1 primer, 1 µl P2 primer, 1 µl of template DNA (50 ng.µl^-1^)). Amplification was performed according to Dvořák et al.^60^. PCR products were purified using E.Z.N.A Cycle Pure Kit (Omega Bio-Tek, Georgia, USA) according to manufacturer´s manual. PCR products were sequenced by Sanger sequencing (Macrogen Europe B.V., Amsterdam, Netherlands, http://dna.macrogen-europe.com) using two additional primers – P5 (5’-TGTACACACCGCCCGTG-3’) and P8 (5’-AAGGAGGTGATCCAGCCACA-3’). Acquired 16S rRNA and 16S-23S ITS sequences were trimmed and assembled in Sequencher 5.0 (Gene Codes Corporation, Ann Arbor, MI, USA). Each clonal culture was represented by one sequence. All obtained 16S rRNA and 16S-23S ITS sequences were identified using the BLAST nucleotide search (http://blast.ncbi.nlm.nih.gov/Blast.cgi). All obtained 16S rRNA and 16S-23S ITS sequences were uploaded to the NCBI database (16S rRNA accession numbers OK173612–23, 16S-23S ITS accession numbers OK173624–35).

### De novo genome sequencing

Two strains were selected for whole genome sequencing–strains A003/A1 (*A. galeatum*) and A004/B2 (*A. antarcticum*). Genomic DNA for whole genome sequencing was extracted from approximately 100 mg of fresh biomass using DNeasy UltraClean Microbial Kit (QuiaGEN, Hilden, Germany) The quality of extracted DNA was checked by agarose gel electrophoresis (1.5% agarose gel, GelRed–Biotium, California, USA) and the DNA was quantified using NanoDrop 1000 (Thermo Fisher Scientific, Waltham, USA). The sequencing was done by commercial Illumina sequencing (Novogene, UK). The whole genome sequences were uploaded to the NCBI database as: BioProject: PRJNA761285, Biosamples: SAMN21250282 (*A. galeatum* A003/A1) and SAMN21250283 (*A. antarcticum* A004/B2), accessions: JAIQZM000000000 and JAIQZN000000000.

### Phylogenetic, phylogenomic reconstructions and ITS secondary structures

NCBI database (https://www.ncbi.nlm.nih.gov/) was used to identify most similar 16S rRNA sequences, using nucleotide BLAST (https://blast.ncbi.nlm.nih.gov/Blast.cgi). Additionally, representative sequences of all sequenced *Phormidium* and *Oscillatoria* species were added to the phylogeny. The multiple sequence alignment was performed in Aliview^63^ using Muscle algorithm 3.8.1551^64^. The Bayesian inference phylogeny was performed in MrBayes 3.2.6^65^ using GTR + I + G model. Two separate runs with four chains (three heated and one cold chain) each were performed for 100 million generations and sampled each 10 000th generation. The analysis was run on the CIPRES Science Gateway v 3.3^66^. All ESS values and PRST values were within the acceptable range. The final tree was constructed using all compatibility consensus rule and 25% of trees were discarded as burn-in. The maximum-likelihood (ML) was performed in IQ-TREE 1.6.5^67^ using GTR + I + G model. The tree topology was tested by 2000 ultrafast bootstrap re-samplings^68^. Maximum parsimony analysis was performed in MEGA X^69^ using all sites and subtree pruning regrafting algorithm. The tree topology was tested using 1000 bootstrap replicates. The tree was edited in FigTree 1.4.4 (http://tree.bio.ed.ac.uk/software/figtree/) and Inkscape^70^. The tree was rooted to the outgroup *Gloeobacter violaceus* (NR074282). Reciprocal p-distance among selected strains related to *Argonema* was computed in MEGA X^69^.

We selected both draft and complete annotated genomes which represent all lineages of cyanobacteria. Moreover, all annotated genome assemblies identified as *Oscillatoria* and *Phormidium* were added. The whole-genome phylogenomic dataset was identified using OrthoFinder 2.3.1^71^ with default settings. The search yielded a multiple sequence alignment with 153 585 amino acid sites. The ML phylogenetic reconstruction was performed in IQ-TREE 1.6.5^67^. The best model was selected using Modeltest implemented in IQ-TREE^72^ based on BIC as follows—LG + F + G4. The tree topology was tested by 2000 ultrafast bootstrap re-samplings^68^. The tree was also edited in FigTree 1.4.4 and Inkscape. To test the hypothesis that A003 and A004 strains belong to two distinct species, average nucleotide identity was estimated in JSpecies 1.2.1 software^36^.

We estimated secondary structures of D1-D1’ and Box B helices using Mfold RNA folding form with default options except for structure draw mode, which was changed to ‘untangle with loop fix’^73^.

### Taxonomy and nomenclature

We combined the monophyletic species concept sensu Johansen & Casamatta^74^ and ANI values^36^ to erect new species. The species and genus description conform to the rules of the International Code of Nomenclature for Algae, Fungi, and Plants (https://www.iapt-taxon.org/nomen/main.php).

### SRA database mining

We used the NCBI SRA database to assess whether *Argonema* DNA was captured in earlier metagenomics studies. We searched through 16S rRNA amplicons, RNA, and whole-metagenome datasets. The fastq files of 1799 amplicon datasets were downloaded using fastq-dump 2.11.0 (SRA tools; https://ncbi.github.io/sra-tools/) with default settings from the SRA archive (https://www.ncbi.nlm.nih.gov/sra). Moreover, one amplicon dataset of soil sample originating in Ladakh (India) was provided by Klára Řeháková (Institute of Hydrobiology ASCR, České Budějovice, Czech Republic). All the sequences were mapped to the reference sequence of 16S rRNA of the strain A003A1 using minimap2 v2.22^75^ with the following command: minimap2 -a -o output.sam A003_A1 database input. The sam files with at least one mapped sequence were converted to fasta using samtools fasta 1.7^76^: samtools fasta -@ 8 -0 output input. The fasta files were searched against the BLAST database of 16S rRNA of the strain A003A1 using blastn^77^. Only hits of 97% similarity and longer than 100 bp were kept. This length was selected because Soergel et al. suggested that short reads with length > 96 bp provide 82–100% as confident identification as the long or full length sequences^78^. The RNAseq (223) and whole-metagenome (191) datasets were downloaded and mapped in the same way as the amplicon datasets. The sam files with coverage of reads > 10 were considered as hits. A diagram of the SRA mining workflow can be found in Supplements (Fig. S6). The world map showing potentional geographical distribution of *Argonema* genus based on metagenomic data was constructed using R software^79^, with packages rnaturalearth v0.1.0^80^ and ggplot2 v.3.3.5^81^, and modified in Inkscape^70^.

## Supplementary Information

Supplementary Information.
