# Supplementary material for: New cyanobacterial genus Argonema is hiding in soil crusts around the world
Source: Sci Rep. 2022 May 3;12:7203. doi: 10.1038/s41598-022-11288-4 (PMC9065122; doi:10.1038/s41598-022-11288-4)

New cyanobacterial genus *Argonema* is hiding in soil crusts around the world

Svatopluk Skoupý, Aleksandar Stanojković, Markéta Pavlíková, Aloisie Poulíčková, Petr Dvořák

**Table S1**: Pairwise genome comparison

| **Tetra** | *Ph..ambiguum.*IAM. M-71 | Ph..LEGE.05292. | A003A1. *A. galeatum* | A004B2. *A. antarcticum* |
| --- | --- | --- | --- | --- |
| *Ph..ambiguum.*IAM. | --- | 0.99572 | 0.89338 | 0.89734 |
| Ph..LEGE.05292. | 0.99572 | --- | 0.91204 | 0.91564 |
| A003A1. *A*. *galeatum* | 0.89338 | 0.91204 | --- | 0.99737 |
| A004B2. *A. antarcticum* | 0.89734 | 0.91564 | 0.99737 | --- |
| **ANIb** |  |  |  |  |
| *Ph..ambiguum*.IAM. | --- | 85.64 | 71.03 | 71.86 |
| Ph..LEGE.05292. | 85.57 | --- | 71.4 | 71.72 |
| A003A1. *A*. *galeatum* | 71.23 | 71.62 | --- | 92.55 |
| A004B2. *A. antarcticum* | 71.73 | 71.85 | 92.16 | --- |
| **ANIm** |  |  |  |  |
| *Ph.ambiguum*.IAM. | --- | 88.18 | 83.63 | 85.73 |
| Ph..LEGE.05292. | 88.19 | --- | 83.98 | 85.29 |
| A003A1. *A*. *galeatum* | 83.67 | 83.9 | --- | 93.74 |
| A004B2. *A. antarcticum* | 85.68 | 85.42 | 93.73 | --- |

**Table S2**: Percent similarity matrix of 16S rRNA of Argonema strains and closely related strains

|  | **Strains** | **1** | **2** | **3** | **4** | **5** | **6** | **7** | **8** | **9** | **10** | **11** | **12** | **13** | **14** | **15** | **16** | **17** | **18** | **19** | **20** | **21** | **22** | **23** | **24** | **25** | **26** | **27** |
| --- | --- | --- | --- | --- | --- | --- | --- | --- | --- | --- | --- | --- | --- | --- | --- | --- | --- | --- | --- | --- | --- | --- | --- | --- | --- | --- | --- | --- |
| **1** | Uncultured *Oscillatoria* sp clone 5cl4; MG250668 |  |  |  |  |  |  |  |  |  |  |  |  |  |  |  |  |  |  |  |  |  |  |  |  |  |  |  |
| **2** | *Oscillatoria princeps* NIVA CYA 150; AB045961 | 96.33 |  |  |  |  |  |  |  |  |  |  |  |  |  |  |  |  |  |  |  |  |  |  |  |  |  |  |
| **3** | Uncultured Oscillatoria sp clone g2; MG250684 | 97.10 | 97.03 |  |  |  |  |  |  |  |  |  |  |  |  |  |  |  |  |  |  |  |  |  |  |  |  |  |
| **4** | *Oscillatoria princeps* CCALA 1115 clone F3; MG255277 | 97.61 | 97.12 | 97.68 |  |  |  |  |  |  |  |  |  |  |  |  |  |  |  |  |  |  |  |  |  |  |  |  |
| **5** | *Oscillatoria princeps* CCALA 1115 clone B1; MG255269 | 97.47 | 97.92 | 97.75 | 98.57 |  |  |  |  |  |  |  |  |  |  |  |  |  |  |  |  |  |  |  |  |  |  |  |
| **6** | *Potamosiphon austaliensis* FHC0914 01; MH047860 | 89.04 | 88.55 | 88.93 | 89.37 | 90.65 |  |  |  |  |  |  |  |  |  |  |  |  |  |  |  |  |  |  |  |  |  |  |
| **7** | *Microseira wollei* K1; MT813673 | 90.20 | 90.86 | 89.79 | 90.60 | 90.71 | 93.25 |  |  |  |  |  |  |  |  |  |  |  |  |  |  |  |  |  |  |  |  |  |
| **8** | *Microseira wollei* K4; MT813676 | 90.16 | 90.83 | 89.86 | 90.56 | 90.71 | 93.23 | 100 |  |  |  |  |  |  |  |  |  |  |  |  |  |  |  |  |  |  |  |  |
| **9** | *Cephalothrix lacustris* CCIBt 3261; KJ994513 | 88.39 | 88.12 | 88.38 | 89.07 | 90.32 | 90.29 | 90.61 | 90.58 |  |  |  |  |  |  |  |  |  |  |  |  |  |  |  |  |  |  |  |
| **10** | *Cephalothrix komarekiana* CCIBt 3277; KJ994514 | 89.02 | 89.18 | 88.91 | 89.77 | 90.87 | 90.44 | 90.41 | 90.38 | 98.75 |  |  |  |  |  |  |  |  |  |  |  |  |  |  |  |  |  |  |
| **11** | *Cephalothrix komarekiana* SAG 75 79; EF654083 | 89.11 | 89.08 | 88.92 | 89.78 | 90.87 | 90.53 | 90.51 | 90.48 | 98.89 | 99.86 |  |  |  |  |  |  |  |  |  |  |  |  |  |  |  |  |  |
| **12** | *Phormidium ambiguum* IAM M 71; AB003167 | 89.48 | 88.70 | 89.20 | 89.99 | 89.43 | 89.01 | 90.23 | 90.19 | 88.94 | 89.37 | 89.55 |  |  |  |  |  |  |  |  |  |  |  |  |  |  |  |  |
| **13** | *Phormidium irriguum* *f minor* ETS 02; FN813342 | 90.04 | 89.46 | 90.44 | 90.28 | 90.13 | 90.90 | 90.73 | 90.70 | 89.63 | 89.53 | 89.71 | 93.05 |  |  |  |  |  |  |  |  |  |  |  |  |  |  |  |
| **14** | *Phormidium cf irriguum* CCALA 759; FN813343 | 90.08 | 89.98 | 90.48 | 90.40 | 90.48 | 91.18 | 91.25 | 91.22 | 89.75 | 90.07 | 89.99 | 93.40 | 97.70 |  |  |  |  |  |  |  |  |  |  |  |  |  |  |
| **15** | *Aerosakkonema funiforme* Lao26; AB686261 | 89.57 | 90.53 | 90.21 | 90.47 | 90.73 | 91.54 | 92.16 | 92.13 | 93.25 | 93.23 | 93.14 | 89.34 | 91.49 | 91.30 |  |  |  |  |  |  |  |  |  |  |  |  |  |
| **16** | *Argonema galeatum* A003_C4 | 87.47 | 87.09 | 88.84 | 88.68 | 89.30 | 88.78 | 86.04 | 86.04 | 88.22 | 88.43 | 88.43 | 85.93 | 87.41 | 88.96 | 90.53 |  |  |  |  |  |  |  |  |  |  |  |  |
| **17** | *Argonema galeatum* A003_C1 | 91.85 | 91.75 | 92.08 | 92.21 | 92.31 | 92.98 | 91.76 | 91.76 | 92.97 | 92.96 | 92.96 | 90.79 | 93.17 | 93.84 | 94.52 | 95.14 |  |  |  |  |  |  |  |  |  |  |  |
| **18** | *Argonema antarcticum* A004_B2 | 92.30 | 92.22 | 92.51 | 92.48 | 92.54 | 93.23 | 92.48 | 92.48 | 92.74 | 92.94 | 92.94 | 91.30 | 93.63 | 94.15 | 94.99 | 93.26 | 98.68 |  |  |  |  |  |  |  |  |  |  |
| **19** | *Argonema antarcticum* A004_D5 | 92.29 | 92.21 | 92.39 | 92.47 | 92.66 | 93.21 | 92.46 | 92.46 | 92.73 | 92.92 | 92.92 | 91.29 | 93.60 | 94.12 | 94.95 | 93.26 | 98.68 | 99.91 |  |  |  |  |  |  |  |  |  |
| **20** | Uncultured Antarctic cyanobacterium clone Fr147; AY151731 | 90.47 | 90.76 | 90.46 | 91.01 | 91.90 | 91.79 | 92.51 | 92.48 | 90.76 | 90.75 | 90.84 | 90.16 | 92.19 | 92.67 | 94.29 | 93.75 | 99.40 | 98.59 | 98.52 |  |  |  |  |  |  |  |  |
| **21** | *Argonema galeatum* A003_B5 | 91.62 | 91.64 | 91.84 | 91.97 | 92.07 | 92.83 | 91.99 | 91.99 | 92.92 | 92.92 | 92.92 | 90.91 | 93.23 | 93.87 | 94.45 | 94.60 | 99.53 | 98.43 | 98.43 | 99.13 |  |  |  |  |  |  |  |
| **22** | *Argonema galeatum* A003_D1 | 92.13 | 92.05 | 92.24 | 92.47 | 92.53 | 93.38 | 92.31 | 92.31 | 93.37 | 93.37 | 93.37 | 91.26 | 93.66 | 94.17 | 95.02 | 95.35 | 99.91 | 98.66 | 98.59 | 99.54 | 99.63 |  |  |  |  |  |  |
| **23** | *Argonema galeatum* A003_B4 | 92.05 | 91.96 | 92.27 | 92.40 | 92.50 | 93.25 | 92.00 | 92.00 | 93.14 | 93.13 | 93.13 | 91.03 | 93.54 | 94.19 | 94.76 | 95.35 | 99.91 | 98.80 | 98.80 | 99.61 | 99.63 | 100 |  |  |  |  |  |
| **24** | *Argonema galeatum* A003_C5 | 91.91 | 91.82 | 92.12 | 92.25 | 92.35 | 93.18 | 91.94 | 91.94 | 93.07 | 93.07 | 93.07 | 91.13 | 93.36 | 93.99 | 94.74 | 95.36 | 99.91 | 98.84 | 98.66 | 99.34 | 99.63 | 99.73 | 100 |  |  |  |  |
| **25** | *Argonema galeatum* A003_D3 | 91.48 | 91.47 | 91.69 | 91.92 | 92.59 | 92.84 | 91.58 | 91.58 | 92.73 | 92.72 | 92.72 | 90.70 | 93.02 | 93.63 | 94.36 | 95.36 | 99.91 | 98.76 | 98.42 | 98.90 | 99.63 | 99.39 | 100 | 99.82 |  |  |  |
| **26** | *Argonema galeatum* A003_C3 | 91.97 | 91.88 | 92.18 | 92.31 | 92.59 | 93.23 | 92.01 | 92.01 | 93.12 | 93.12 | 93.12 | 91.09 | 93.42 | 94.03 | 94.78 | 95.36 | 99.91 | 98.76 | 98.67 | 99.35 | 99.63 | 99.74 | 100 | 99.82 | 100 |  |  |
| **27** | *Argonema galeatum* A003_A1 | 92.07 | 91.98 | 92.29 | 92.42 | 92.52 | 93.27 | 92.00 | 92.00 | 93.15 | 93.15 | 93.15 | 91.05 | 93.45 | 94.10 | 94.77 | 95.36 | 99.91 | 98.81 | 98.81 | 99.52 | 99.63 | 100 | 100 | 100 | 100 | 100 |  |
| **28** | *Argonema galeatum* A003_A3 | 91.91 | 91.92 | 92.25 | 92.39 | 92.51 | 93.18 | 92.34 | 92.34 | 93.50 | 93.61 | 93.61 | 91.19 | 93.83 | 94.28 | 95.22 | 95.36 | 99.90 | 98.74 | 98.74 | 99.49 | 99.61 | 100 | 100 | 100 | 100 | 100 | 100 |

**Table S3**: Sequences from metagenomic datasets, their geographical location and annual precipitation.

| SRA accession | Habitat | Geographical location | Mean annual precipitation | Citation |
| --- | --- | --- | --- | --- |
| Amplicon sequencing SRA data | | |  |  |
| **DRR148102** | soil crust | Svalbard | 580 mm | (Unpublished) Glacier metagenome, Kreidenweis Group, Atmospheric Science, Colorado State University (2018) |
| **ERR3220011** | desert soil crust | Oman, Haat | 180 mm | R. M. M. Abed, A. Tamm, C Hassenrück et al. (2019) Habitat-dependent composition of bacterial and fungal communities in biological soil crusts from Oman. Sci Rep 9, 6468 |
| **ERR3427488** | soil, Hamada socaria shrub | Israel, Negev desert | 185 mm | (Unpublished) Commercially available reverse transcription enzyme and temperature comparison in high throughput sequencing of bacterial ribosomal 16S rRNA, Ben Gurion University of The Negev (2019) |
| **ERR3866785** | soil crust | Israel, Negev desert | 185 mm | (Unpublished) Metabolic versatility and different drought survival strategies in biologic soil crust microorganisms revealed by population-resolved metagenomics, University of Vienna (2020) |
| **SRR11485336** | desert soil crust | China, Tengger dessert | 190 mm | (Unpublished) Cyanobacteria 16S rDNA sequencing, Northwest Institute of Eco-Environment and Resources, Chinese Academy of Sciences (2020) |
| **SRR11545584** | dessert soil | USA, Joshua Tree National Park | 8.6 mm | (Unpublished) Soil crust metagenome Raw sequence reads, University of California Riverside (2020) |
| **SRR11801452** | steppe soil crust | USA, Reynolds Creek | 57.8 mm | (Unpublished) Biological soil crust communities along an elevational gradient in Reynolds Creek CZO, Idaho State University (2020) |
| **SRR12233654** | soil crust | Germany, Speyer | 305 mm | (Unpublished) The Effect of Agricultural Management on the Microbiome of Biocrusts and Bulk Soil, mothur (2020) |
| **SRR13170662** | soil crust | Israel, Negev dessert | 185 mm | (Unpublished) 16S sequences from biological soil crust samples collected in a hyper arid desert, Ben Gurion University, 1.12.2020 |
| **SRR13757133** | soil | China, Tengger dessert | 190 mm | (Unpublished) Microbial community change in near space, Chinese Academy of Sciences (2021) |
| **SRR13759753** | soil | China, Tengger dessert | 190 mm | (Unpublished) Archaea diversity of algal crusts, Chinese Academy of Sciences (2021) |
| **SRR14570662** | soil crust | China, Zhangye | 1350 mm | (Unpublished) Y. Wang, Bacterial community in a desert biological soil crusts, Inner Mongolia Normal University (2021) |
| **SRR1557188** | early crust | China, Gurbantunggut desert | 70 - 180 mm | (Unpublished) China Gurbantunggut Desert Cyanobacteria Metagenome, Xinjiang Institute of Ecology and Geography (2014) |
| **SRR2917986** | soil crust | USA, Utah, Moab | 60 mm | (Unpublished) E. Couradeau, Biological Soil Crust Raw sequence reads, Arizona State University (2015) |
| **SRR3669940** | Soil crust | Germany, Goessenheim | 740 mm | L. Briegel-Williams, J. Loewen-Schneider, S. Maier, B. Büdel (2016) Cyanobacterial diversity of western European biological soil crusts (BSCs) along a latitudinal gradient, FEMS Microbiology Ecology, 92(10):fiw157 |
| **SRR3669943** | Soil crust | Austria, Hohe Tauern | 1133 mm | L. Briegel-Williams, J. Loewen-Schneider, S. Maier, B. Büdel (2016) Cyanobacterial diversity of western European biological soil crusts (BSCs) along a latitudinal gradient, FEMS Microbiology Ecology, 92(10):fiw157 |
| **SRR3669944** | Soil crust | Spain, Tabernas | 109 mm | L. Briegel-Williams, J. Loewen-Schneider, S. Maier, B. Büdel (2016) Cyanobacterial diversity of western European biological soil crusts (BSCs) along a latitudinal gradient, FEMS Microbiology Ecology, 92(10):fiw157 |
| **SRR3669952** | soil | Sweden, Oeland | 390 mm | L. Briegel-Williams, J. Loewen-Schneider, S. Maier, B. Büdel (2016) Cyanobacterial diversity of western European biological soil crusts (BSCs) along a latitudinal gradient, FEMS Microbiology Ecology, 92(10):fiw157 |
| **SRR4277938** | soil crust | Israel, Negev region | 185 mm | (Unpublished) S. Laor, Soil crust metagenome Raw sequence reads, Ben Gurion University (2016) |
| **SRR4288782** | soil | USA, Arizona | 38 mm | S. V. Ayuso, A. G. Silva, C. Nelson, N. Barger, G. Garcia-Pichel (2016) Microbial nursery production of high-quality biological soil crust biomass for restoration of degraded dryland soils, Applied and Environmental Microbiology, 83(3) |
| **SRR4407974** | soil | China, Tengger dessert | 190 mm | (Unpublished) Y. Liu, Paddy soil Raw sequence reads, Northwest Institute of Eco-Environment and Resources (2016) |
| **SRR5108067** | soil crust | Australia, Perenjori | 328 mm | (Unpublished) T. Santini, Environmental samples Raw sequence reads, The University of Queensland (2016) |
| **SRR5382147** | soil crust | Svalbard | 580 mm | I. S. Pessi, E. Pushkareva, Y. Lara, F. Borderie, A. Wilmottte, J. Elster (2019) Marked Succession of Cyanobacterial Communities Following Glacier Retreat in the High Arctic, Microb. Ecol. 77(1):136-147 |
| **SRR5431209** | desert soil crust | Israel, Negev dessert | 185 mm | N. Wieler, H. Ginat, O. Gillor, R. Angel (2019) The origin and role of biological rock crusts in rocky desert weathering, Biogeosciences, 16,1133-1145 |
| **SRR5576303** | soil crust | China, Tibet Plateau | 490 mm | (Unpublished) H. Li, Phototrophic and diazotrophic diversity in crustal communities of Tibetan Plateau, Chinese Academy of Sciences (2017) |
| **SRR5985851** | soil crust | USA, Mojave Desert | 14 mm | R. Mogul et al. (2017) Microbial Community and Biochemical Dynamics of Biological Soil Crusts across a Gradient of Surface Coverage in the Central Mojave Desert, Front Microbiol, 2017;8:1974 |
| **SRR5998466** | desert soil crust | China, Zhongwei | 245 mm | (Unpublished) T. Zhang, Soil crust metagenome Raw sequence reads, Institute of Medicinal Biotechnology, Chinese Academy of Medical Sciences (2017) |
| **SRR6222309** | soil crust | Svalbard | 580 mm | L. Williams, N. Borchhardt, C. Colesie, C. Baum, K. Komsic-Buchmann, M. Rippin et al. (2017) Biological soil crusts of Arctic Svalbard and of Livingston Island, Antarctica. Polar Biol. 40: 399Ð411. |
| **SRR6222321** | soil crust | Antarctica, Livingston island | 380 mm | L. Williams, N. Borchhardt, C. Colesie, C. Baum, K. Komsic-Buchmann, M. Rippin et al. (2017) Biological soil crusts of Arctic Svalbard and of Livingston Island, Antarctica. Polar Biol. 40: 399Ð411. |
| **SRR7343078** | soil crust | Australia, Diamantina NP | 141 mm | (Unpublished) D. Elliott, Microbial diversity in dryland soils, University of Derby (2018) |
| **SRR8133478** | soil | Spain | NA | (Unpublished) R. Thombre, Metagenome of Laguna de Pena Hueca, Spain, Modern College,Shivajinagar (2018) |
| **SRR8380812** | Soil | China | NA | (Unpublished) Y. G. Su, Microbe sample from Biological soil crusts, Fujian normal university (2018) |
| **SRR8445684** | soil crust | USA, New Mexico | 35 mm | J. B. Rakes, A. Giraldo-Silva, C. Nelson, N. Barger, F. Garcia-Pichel (2019) Optimizing production of nursery-based biological soil crusts for restoration of arid land soils, Applied and Environmental Microbiology, 85(15) |
| **SRR8445704** | soil crust | USA, Utah, Great salt ake | 19 mm | J. B. Rakes, A. Giraldo-Silva, C. Nelson, N. Barger, F. Garcia-Pichel (2019) Optimizing production of nursery-based biological soil crusts for restoration of arid land soils, Applied and Environmental Microbiology, 85(15) |
| **SRR9106059** | soil | USA, Mojave Desert | 14 mm | (Unpublished) N. Pombubpa, Biological soil crust microbiome in Mojave Desert, USA, University of California Riverside (2019) |
| **SRR9686857** | desert crust | China, Inner Mongolia | 190 mm | (Unpublished) H. Zhou, bacterial sequencing of different developmental stages of soil crusts, Chinese Academy of Forestry (2019) |
| **SRR9997735** | soil crust | Svalbard | 580 mm | E. Pushkareva, A. Wilmotte, K. Láska, J. Elster (2019) Comparison of Microphototrophic Communities Living in Different Soil Environments in the High Arctic, Frontiers in Ecology and Evolution, 7:393 |
| Metagenomic sequencing SRA data | | |  |  |
| **SRR11829740** | soil crust | USA, California | 8.6 mm | (Unpublished) JGI, Biological soil crust microbial communities from Mojave Desert, California, United States - LAC_S1, University of California (2020) |
| **SRR5855411** | soil crust | USA, Utah, Moab | 59.2 mm | (Unpublished) Ulas Karaoz, Late stage wet biocrust from a biocrust gradient ranging from least to most mature as follows 'early, early-mid, late-mid, late' collected dry from a bundle, Lawrence Berkeley National Laboratory (2017) |
| **SRR12951525** | soil crust | USA, California | 7.7 mm | (Unpublished) JGI, Hormoscilla sp. CMT-3BRIN-NPC48 and co-occurring heterotrophs from soil crust from Clark Mountains Wilderness, California, United States - 20190625_12A, University of California (2020) |
| **SRR13608738** | soil crust | USA, Utah, Moab | 59.2 mm | (Unpublished) M. V. Goethem, Metagenome or environmental sample from soil crust metagenome, Lawrence Berkeley National Laboratory (2021) |
| Partial 16SrRNA NCBI sequences | | |  |  |
| **AY151724** | lake microbial mat | Anrarctica, Victoria land, Taylor valley | 112 mm | A. Taton, S. Grubisic, E. Brambilla, R. De Wit, A. Wilmotte (2003) Cyanobacterial diversity in natural and artificial microbial mats of Lake Fryxell (McMurdo Dry Valleys, Antarctica): a morphological and molecular approach, Appl. Environ. Microbiol. 69 (9), 5157-5169 |
| **AY151731** | lake microbial mat | Anrarctica, Victoria land, Taylor valley | 113 mm | A. Taton, S. Grubisic, E. Brambilla, R. De Wit, A. Wilmotte (2003) Cyanobacterial diversity in natural and artificial microbial mats of Lake Fryxell (McMurdo Dry Valleys, Antarctica): a morphological and molecular approach, Appl. Environ. Microbiol. 69 (9), 5157-5169 |
| **AY151731** | lake microbial mat | Anrarctica, Victoria land, Taylor valley | 114 mm | A. Taton, S. Grubisic, E. Brambilla, R. De Wit, A. Wilmotte (2003) Cyanobacterial diversity in natural and artificial microbial mats of Lake Fryxell (McMurdo Dry Valleys, Antarctica): a morphological and molecular approach, Appl. Environ. Microbiol. 69 (9), 5157-5169 |
| **JN814150** | soil | Anrarctica, Victoria land, Taylor valley | 115 mm | A. B. Michaud, M. Sabacka, J. C. Priscu (2012) Cyanobacterial diversity across landscape units in a polar desert: Taylor Valley, Antarctica, FEMS Microbiol. Ecol. 82 (2), 268-278 |
| **JN814284** | soil | Anrarctica, Victoria land, Taylor valley | 116 mm | A. B. Michaud, M. Sabacka, J. C. Priscu (2012) Cyanobacterial diversity across landscape units in a polar desert: Taylor Valley, Antarctica, FEMS Microbiol. Ecol. 82 (2), 268-278 |
| **KM112146** | lake microbial mat | Antarctica, McMurdo Dry Valley | 10 mm | (Unpublished) L. Zhang, A. D. Jungblut, I. Hawes, T. J. Mackey, D. Y. Sumner, D. T. Andersen Cyanobacterial diversity in benthic mats of the McMurdo Dry Valley Lakes, Antarctica |
| **HQ827314** | lake microbial mat | Antarctica, King George island | 350 mm | (Unpublished) C. Callejas, E. M. de Souza, S. Batista (2010) Molecular Microbiology Unit, Instituto de Investigaciones Biologicas Clemente Estable (IIBCE), Uruguay |
| **HQ827346_** | lake microbial mat | Antarctica, King George island | 350 mm | (Unpublished) C. Callejas, E. M. de Souza, S. Batista (2010) Molecular Microbiology Unit, Instituto de Investigaciones Biologicas Clemente Estable (IIBCE), Uruguay |
| **HQ827360** | lake microbial mat | Antarctica, King George island | 350 mm | (Unpublished) C. Callejas, E. M. de Souza, S. Batista (2010) Molecular Microbiology Unit, Instituto de Investigaciones Biologicas Clemente Estable (IIBCE), Uruguay |
| **HQ827398** | lake microbial mat | Antarctica, King George island | 350 mm | (Unpublished) C. Callejas, E. M. de Souza, S. Batista (2010) Molecular Microbiology Unit, Instituto de Investigaciones Biologicas Clemente Estable (IIBCE), Uruguay |
| **HQ827660** | lake microbial mat | Antarctica, King George island | 350 mm | (Unpublished) C. Callejas, E. M. de Souza, S. Batista (2010) Molecular Microbiology Unit, Instituto de Investigaciones Biologicas Clemente Estable (IIBCE), Uruguay |
| **HQ827661** | lake microbial mat | Antarctica, King George island | 350 mm | (Unpublished) C. Callejas, E. M. de Souza, S. Batista (2010) Molecular Microbiology Unit, Instituto de Investigaciones Biologicas Clemente Estable (IIBCE), Uruguay |
| **DQ264199** | freshwater lake | Luxembourg | 875 mm | R. Willame, C. Boutte, S. Grubisic, A. Wilmotte, J. Komarek, L. Hoffmann (2006) Morphological and molecular characterization of planktonic cyanobacteria from Belgium and Luxembourg, J. Phycol. 42 (6), 1312-1332 |
| **HQ189086** | alpine soil | Nepal, Anapurna range | 1154 mm | S. K. Schmidt, R. C. Lynch, A. J. King, D. Karki, M. S. Robeson, L. Nagy, M. W. Williams, M. S. Mitter, K. R. Freeman (2011) Phylogeography of microbial phototrophs in the dry valleys of the high Himalayas and Antarctica, Proc. Biol. Sci. 278 (1706), 702-708 |
| **LN835824** | soil – halophyte roots | Pakistan, Punjab | 375 mm | (Unpublished) S. Mukhtar, K. A. Malik, M. S. Mirza, S. Mehnaz (2015) Microbial diversity and metagenomic analysis of the rhizosphere of Haloxylon recurvum growing under highly saline conditions |
| Additional sequences | |  |  |  |
| **prov. by Klara Rehakova** | soil | Inida, Ladakh | 740 mm | (Unpublished) K. Rehakova, nstitute of Hydrobiology ASCR, Ceske Budejovice, Czech Republic |

**Climatic data were aquired from:** **Copyright ©**[Time and Date AS](https://www.timeanddate.com/company/)**1995-2021**, URL: https://www.timeanddate.com (2021)

**Fig. S1**: Microphotographs of original environmental samples


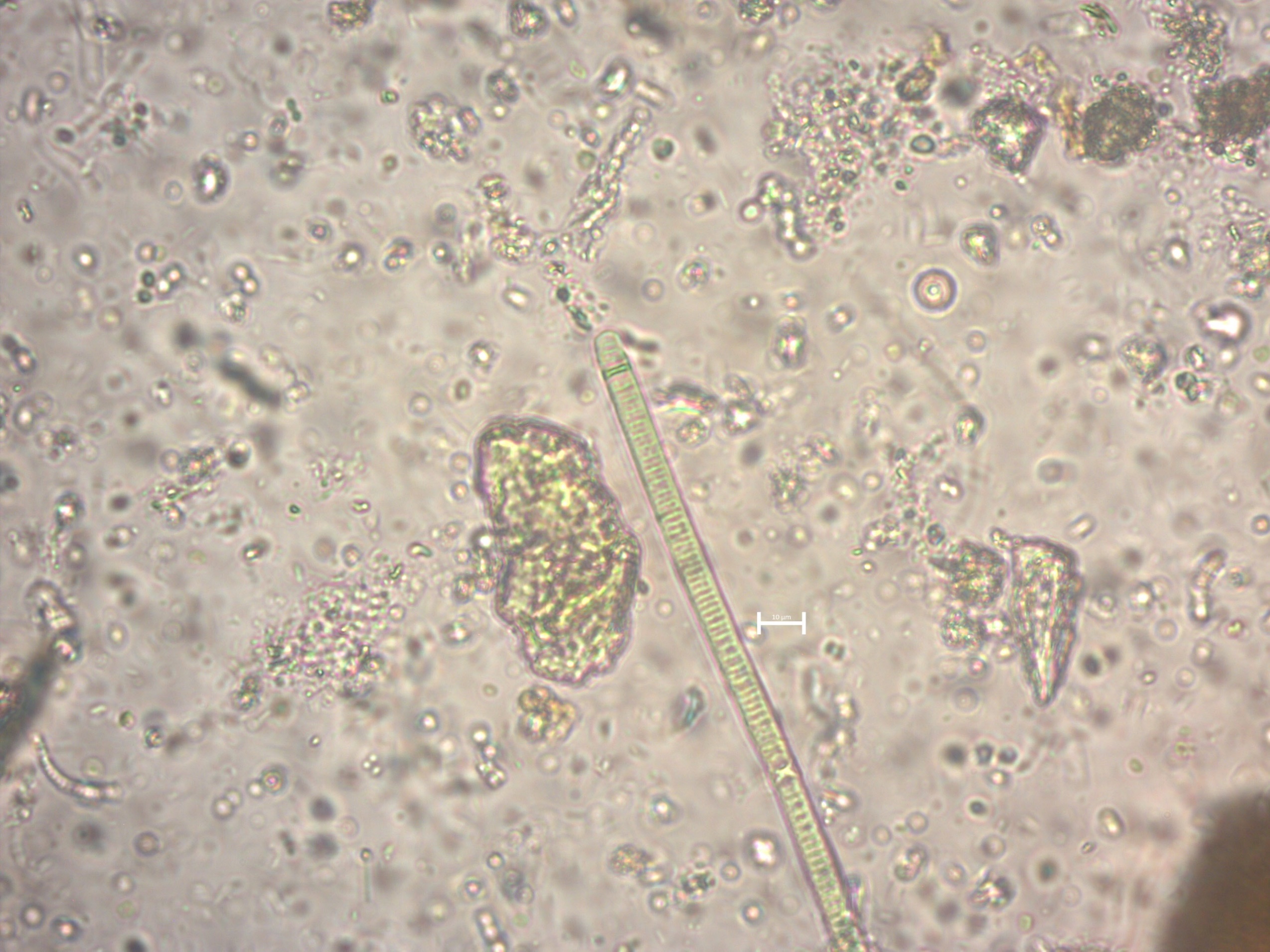

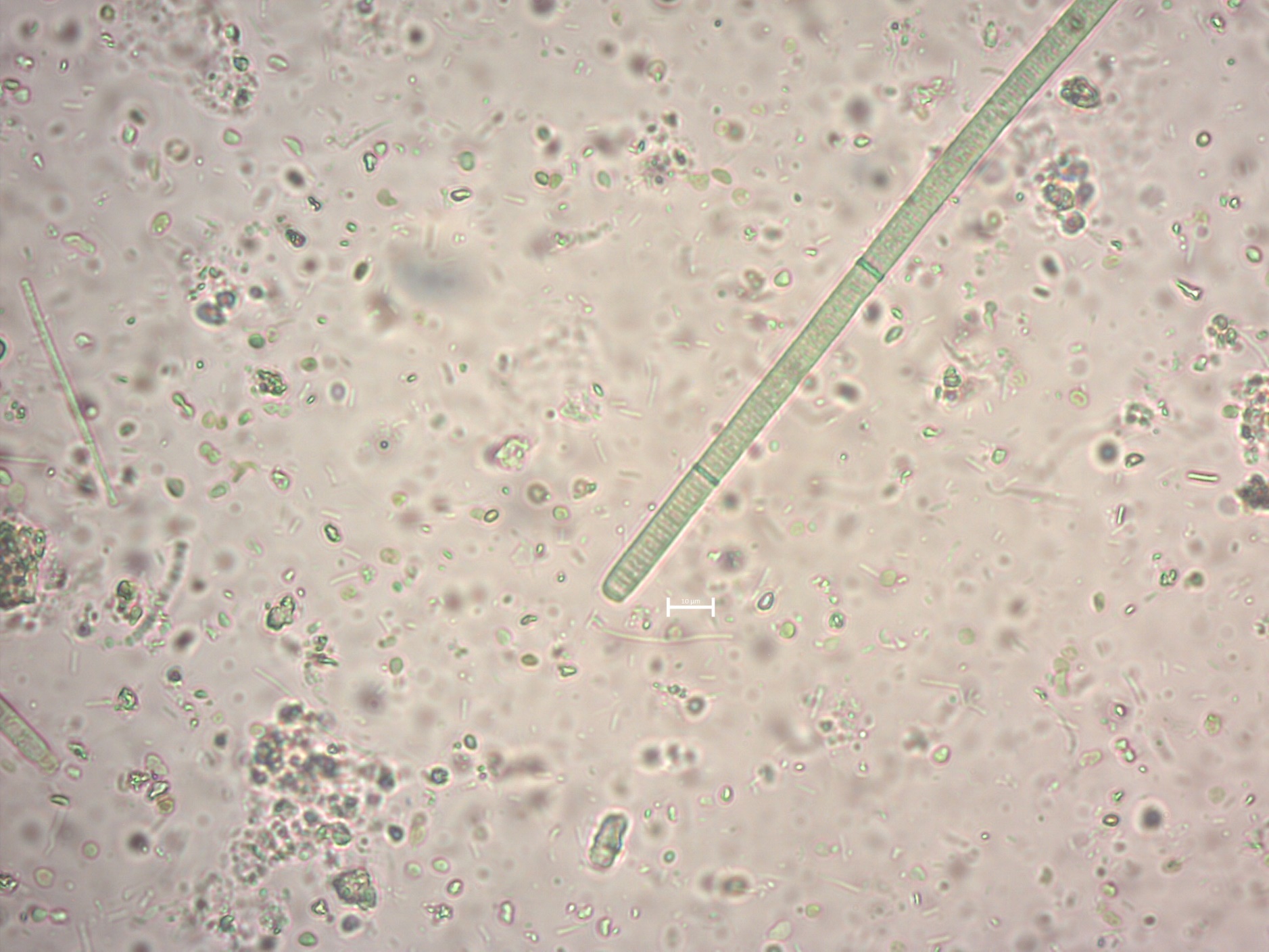

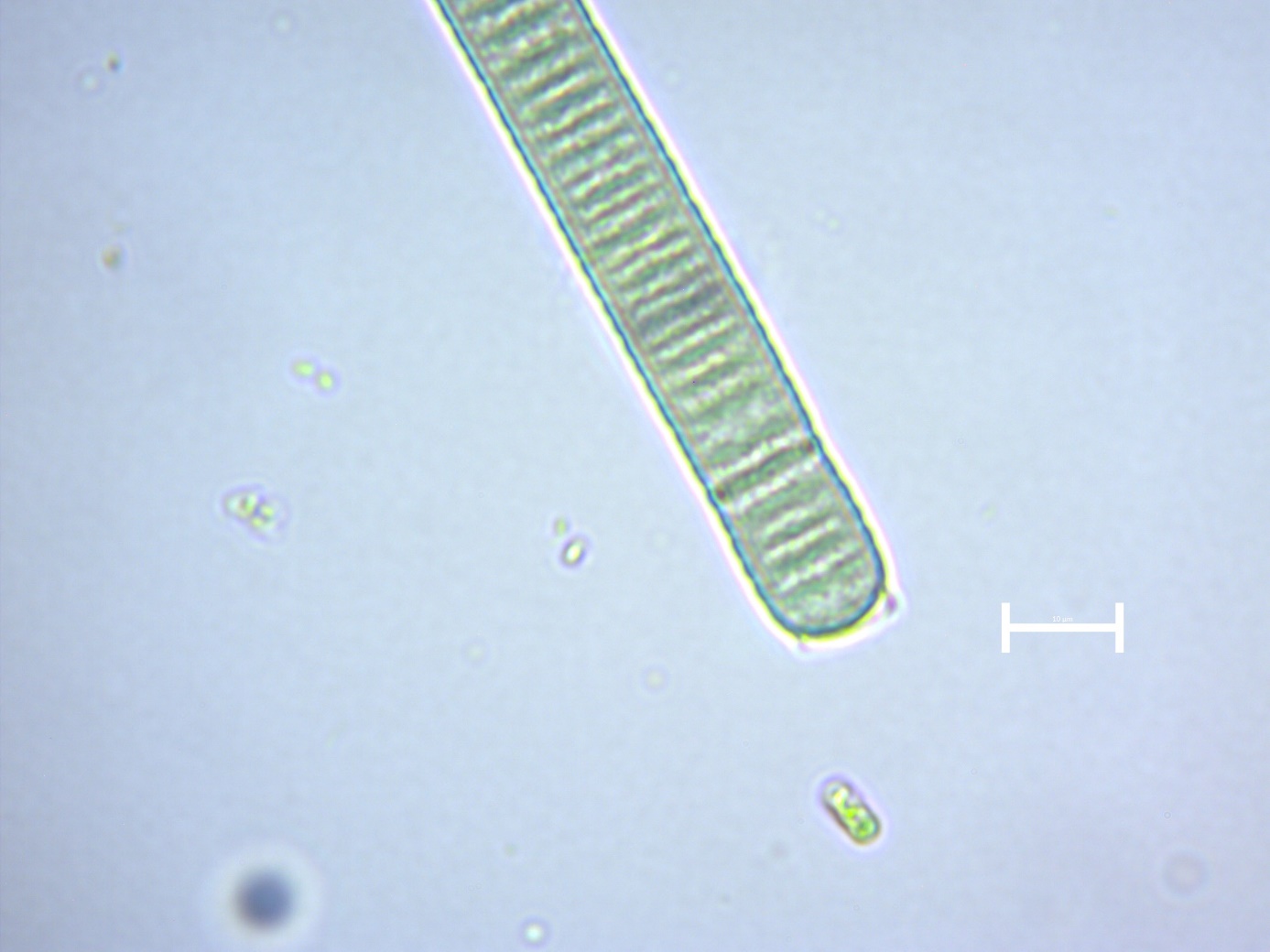

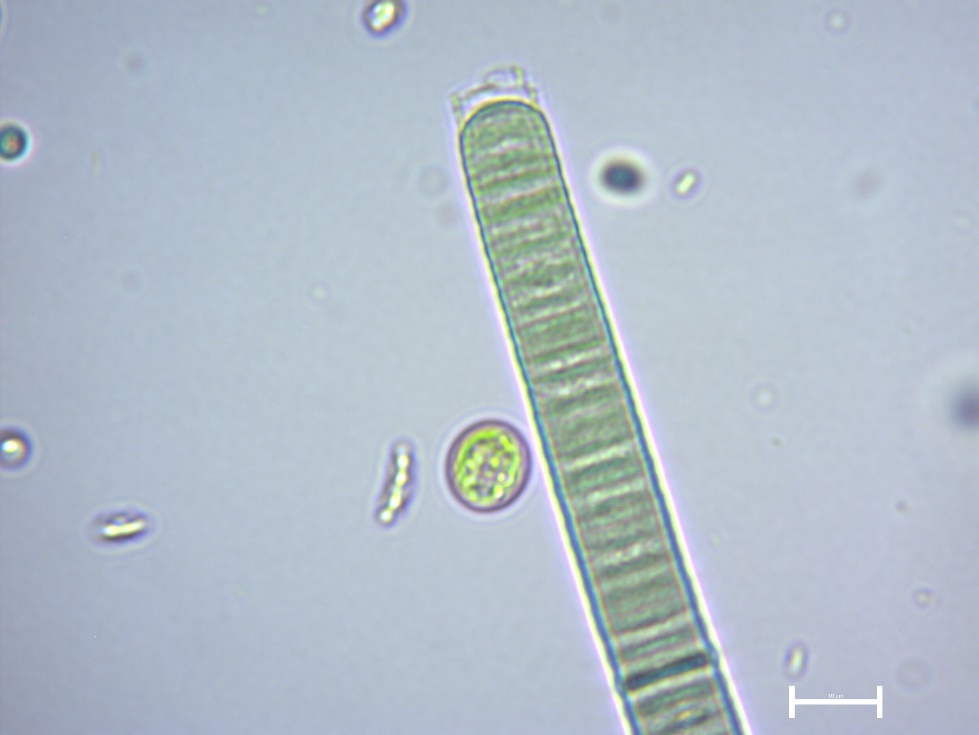


**a**

**b**

**c**

**d**

**Fig S1a, b**: Microphotographs of *D. galeatum* fillaments from original environmental sample A003. **Fig S1c, d**: Microphotographs of *D. antarcticum* fillaments from the original environmental sample A004

**Fig. S2**: Two disticnt insertions/deletions present in 16S-23S ITS sequences of *Argonema* strains


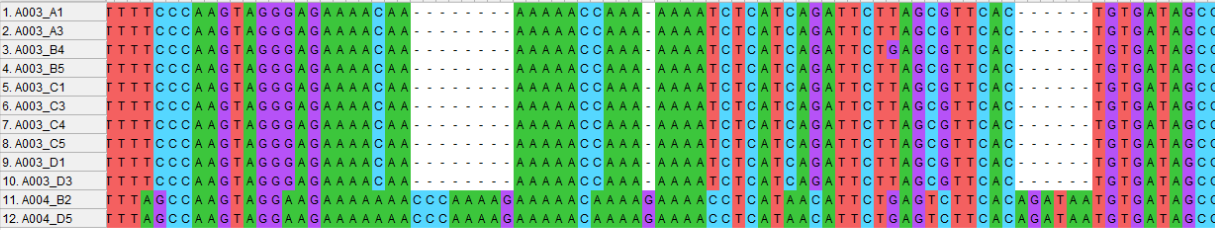


**A003** strains – *Argonema galeatum*, **A004** strains – *Argonema antarcticum*

**Fig. S3**: A reconstruction of 16S-23S ITS secondary structures of *A. galeatum* and *A. antarcticum*; D1-D1' (a) and Box-B (b) helices are shown


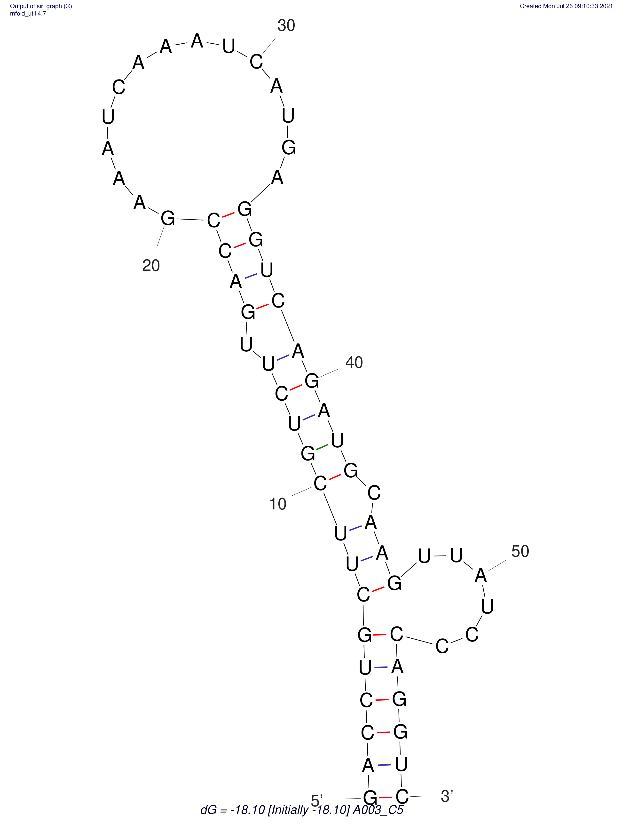

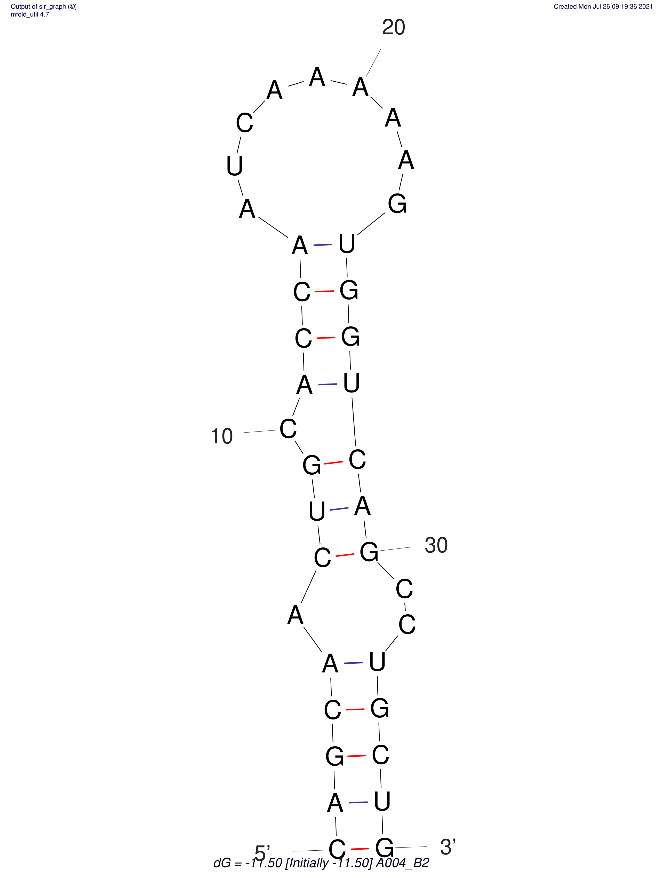


**b**

**a**

**Fig. S4**: Partial 16S rRNA maximum likelihood tree. Nodes with bootstrap support of 99 and 100 are marked by a star.


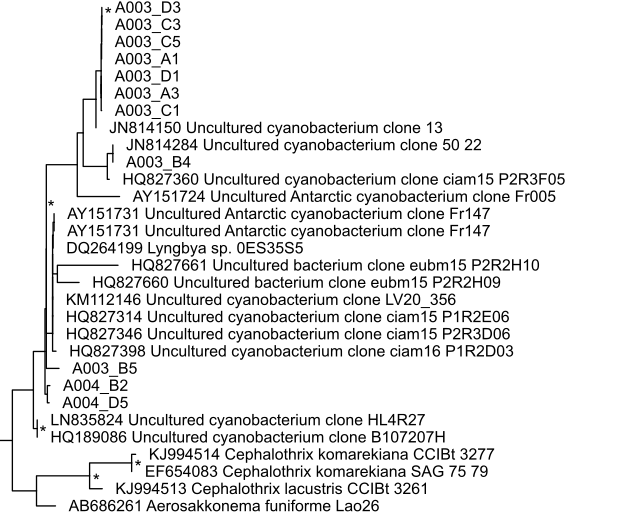


**A003** strains – *Argonema galeatum*, **A004** strains – *Argonema antarcticum*

**Fig. S5**: NCBI SRA data mining workflow diagram


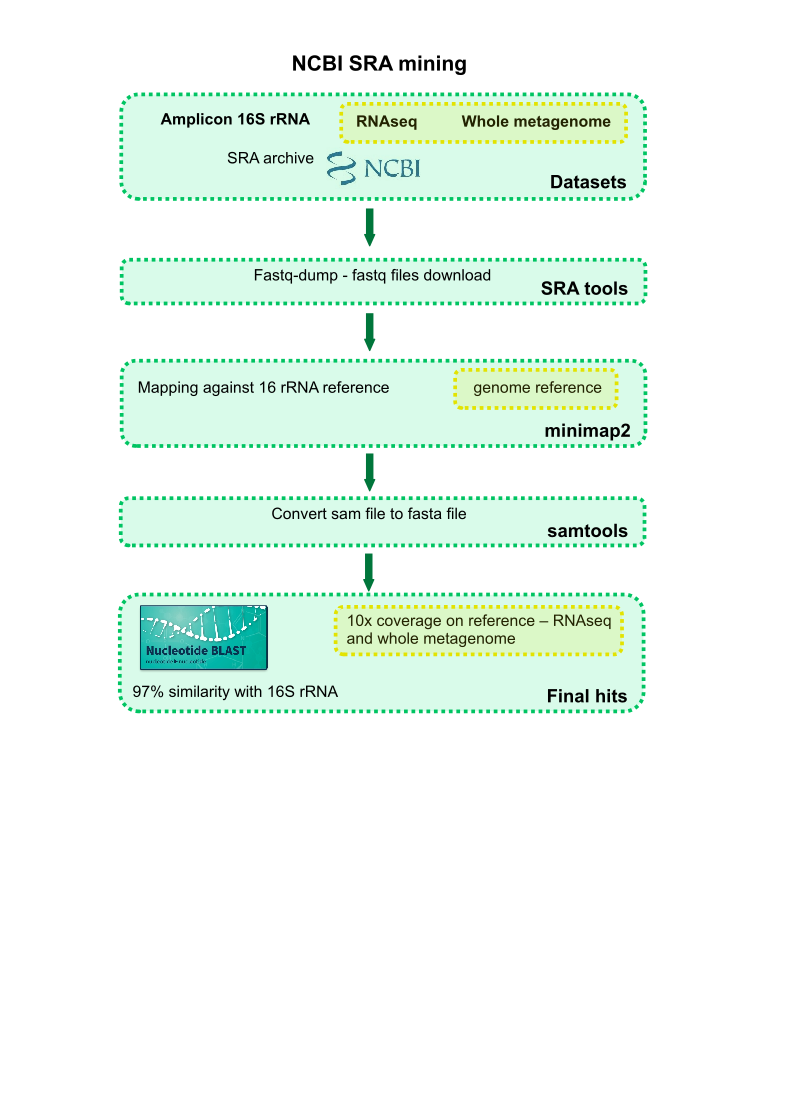

Supplement: Supplementary file 1 — Supplementary Information. [file 41598_2022_11288_MOESM1_ESM.docx]
